# Correlation of gene expression and associated mutation profiles of *APOBEC3A*, *APOBEC3B*, *REV1*, *UNG*, and *FHIT* with chemosensitivity of cancer cell lines to drug treatment

**DOI:** 10.1186/s40246-018-0150-x

**Published:** 2018-04-11

**Authors:** Suleyman Vural, Richard Simon, Julia Krushkal

**Affiliations:** Computational and Systems Biology Branch, Biometric Research Program, Division of Cancer Treatment and Diagnosis, National Cancer Institute, 9609 Medical Center Dr, Rockville, MD 20850 USA

**Keywords:** APOBEC mutagenesis, Cell line, Chemosensitivity, Gene expression

## Abstract

**Background:**

The *APOBEC* gene family of cytidine deaminases plays important roles in DNA repair and mRNA editing. In many cancers, APOBEC3B increases the mutation load, generating clusters of closely spaced, single-strand-specific DNA substitutions with a characteristic hypermutation signature. Some studies also suggested a possible involvement of APOBEC3A, REV1, UNG, and FHIT in molecular processes affecting APOBEC mutagenesis. It is important to understand how mutagenic processes linked to the activity of these genes may affect sensitivity of cancer cells to treatment.

**Results:**

We used information from the Cancer Cell Line Encyclopedia and the Genomics of Drug Sensitivity in Cancer resources to examine associations of the prevalence of APOBEC-like motifs and mutational loads with expression of *APOBEC3A*, *APOBEC3B*, *REV1*, *UNG*, and *FHIT* and with cell line chemosensitivity to 255 antitumor drugs. Among the five genes, *APOBEC3B* expression levels were bimodally distributed, whereas expression of *APOBEC3A*, *REV1*, *UNG*, and *FHIT* was unimodally distributed. The majority of the cell lines had low levels of *APOBEC3A* expression. The strongest correlations of gene expression levels with mutational loads or with measures of prevalence of APOBEC-like motif counts and kataegis clusters were observed for *REV1*, *UNG*, and *APOBEC3A*. Sensitivity or resistance of cell lines to JQ1, palbociclib, bicalutamide, 17-AAG, TAE684, MEK inhibitors refametinib, PD-0325901, and trametinib and a number of other agents was correlated with candidate gene expression levels or with abundance of APOBEC-like motif clusters in specific cancers or across cancer types.

**Conclusions:**

We observed correlations of expression levels of the five candidate genes in cell line models with sensitivity to cancer drug treatment. We also noted suggestive correlations between measures of abundance of APOBEC-like sequence motifs with drug sensitivity in small samples of cell lines from individual cancer categories, which require further validation in larger datasets. Molecular mechanisms underlying the links between the activities of the products of each of the five genes, the resulting mutagenic processes, and sensitivity to each category of antitumor agents require further investigation.

## Background

APOBEC3A and APOBEC3B (apolipoprotein B mRNA-editing enzymes 3A and 3B, catalytic polypeptide-like) are cytosine deaminases from the AID/APOBEC family, members of which play important roles in host immunity against pathogens [1, 2]. The activity of multiple members of the AID/APOBEC family including APOBEC3A but not APOBEC3B has also been linked to epigenetic processes involving DNA demethylation via deamination of 5-hydroxymethyl-cytozine (5-hmC) to 5-hydroxymethyl-uracil (5-hmU) [1, 3, 4]. APOBEC3B is an endogenous mutagen which generates DNA substitutions, most frequently C to T, via a process that involves cytosine to uracil deamination of single-stranded DNA, most commonly in the 5′-TCW-3′ (where W is either A or T) sequence context [2]. In multiple human cancer categories, increased *APOBEC3B* gene expression has been associated with genome-wide hypermutation and with kataegis, a mutagenic process that generates clusters of closely spaced, single-strand-specific DNA substitutions, which are predominantly C to T [5, 6]. Clusters of APOBEC3B mutations are often localized at breakpoints of chromosomal rearrangements [2]. Increased *APOBEC3B* gene expression, germline polymorphisms in the *APOBEC3* genome region, and higher degree of abundance of APOBEC3B mutational signatures have been associated with increased cancer risk and patient survival [5, 7].

APOBEC3B mutagenesis has a characteristic pattern of mutational specificity. It is most commonly represented by the 5′-T(C>T)W-3′ sequence motif [8], where “>” indicates the C to T substitution, and W is an [A or T]. This hypermutation pattern and high mRNA expression levels of *APOBEC3B* have been found in several cancer types [9, 10]. Additional mutation patterns have also been reported for APOBEC3B, although some of these patterns may also be attributed to other APOBEC family members [6, 7, 10, 11]. According to various reports, in addition to the C>T transitions, these patterns may include possible C>G and, in some specific cancer types such as ovarian carcinomas, C>A transversions, as well as a possible 5′-TC(A or G)-3′ sequence context, so that possible mutational motifs could be represented as 5′-T(C>K)W-3′, 5′-T(C>D)R-3′, or 5′-T(C>D)D-3′, where K is [G or T], W is [A or T], R is [A or G], and D is [A or G or T] according to the IUB-IUPAC ambiguity codes [6–8, 11–13]. Below, we present these sequence motifs in the 5′ to 3′ direction as T(C>K)W, T(C>D)R, and T(C>D)D.

While APOBEC3B plays a prominent role in cancer mutagenesis, several other AID/APOBEC family members also have mutagenic roles and affect DNA integrity [9, 14]. Most of them have separate distinct specificities for genome sequence context [2, 8–10, 15, 16]. However, a possible overlap between the activities of APOBEC3B and APOBEC3A has not been fully resolved. The *APOBEC3A* gene is located in proximity to *APOBEC3B* in the *APOBEC* genomic cluster in the chromosomal region 22q13.1 [7]. An *APOBEC3A-APOBEC3B* fusion transcript may be produced due to a germline deletion polymorphism, which results in the complete loss of the coding part of the *APOBEC3B* gene and abolishes *APOBEC3B* gene expression; this deletion polymorphism produces a fusion product of the *APOBEC3A* gene with the 3′-UTR of *APOBEC3B* gene, and it has been associated with an increased risk of several types of cancer [7, 17]. The evidence for a mutagenic role of APOBEC3A so far has been less conclusive than that of APOBEC3B [12, 18]. However, a number of studies suggested that APOBEC3A also acts as an endogenous mutagen that can produce genomic damage, with a mutation signature that may be distinguishable to some extent from that of APOBEC3B [7, 13, 19–25]. In addition to mutagenesis linked to DNA deamination of single-stranded DNA, both APOBEC3B and APOBEC3A can bind RNA, and APOBEC3A has been reported to be involved in both C to U and G to A RNA editing [16, 26].

Based on the strong evidence for APOBEC-associated mutagenesis in a variety of cancer types, it is important to learn whether such mutagenic processes may affect cancer response to therapy, in order to exploit potential pathways involved in sensitivity and to avoid potential mechanisms of resistance. To date, the effect of APOBEC3B-like mutagenic processes on therapeutic response has not been fully understood, with several reports of divergent directions of association. Some studies suggested a potential role of APOBEC mutagenesis in tumor resistance to therapy, with a possible resistance mechanism explained by increased tumor heterogeneity when APOBEC3B activity is elevated [18]. Clinical studies and an analysis of murine xenograft models found an association of increased *APOBEC3B* mRNA expression levels with tamoxifen resistance in estrogen receptor-positive (ER^+^) breast cancer [18]. In an analysis of 30 human cell lines, expression levels of the *APOBEC3B* gene were associated with resistance to vinblastine, topotecan, paclitaxel, mitoxantrone, mitomycin C, etoposide, and doxorubicin [27]. In contrast, a study of bladder cancer patients from the Cancer Genome Atlas (TCGA) demonstrated improved survival of those patients who had elevated numbers of APOBEC signature mutations [7]. Experimental in vitro overexpression of *APOBEC3B* in the 293-A3B and 293-GFP cell lines with inactivated p53 resulted in an increase in APOBEC mutagenesis and kataegic events, which were accompanied by cell hypersensitivity to small-molecule DNA damage response inhibitors including ATR (VX-970 and AZD673), CHEK1 (SAR020106), CHEK2 (CCT241553), PARP (olaparib and BMN-673), and WEE1 (AZD1775) inhibitors, as well as by sensitivity to combinations of cisplatin/ATR inhibitor, ATR/PARP inhibitor, and PARP/WEE1 inhibitor [28]. Increased *APOBEC3B* expression in breast cell lines was also correlated with sensitivity to the CHEK1 inhibitor CCT244747 [29]. In contrast, *APOBEC3B* or *APOBEC3A* expression levels were not significantly correlated with sensitivity to any drugs in breast cancer cell lines from the Genomics of Drug Sensitivity in Cancer (GDSC, or GDS1000) dataset [30]; however, they were associated with sensitivity to 38 and 16 agents, respectively, in a joint analysis of all cancer types [31].

At the molecular level, APOBEC3B hypermutation activity has been reported to have a synergistic effect with the absence of the uracil-specific uracil DNA glycosylase (UNG) and to involve molecular steps that require the activity of the translesion synthesis DNA polymerase REV1 [8, 20, 22, 24]. APOBEC mutagenesis may also be increased in case of reduced expression or the loss of protein activity of the tumor suppressor fragile histidine triad protein (FHIT), and higher levels of APOBEC mutagenesis were observed in TCGA lung adenocarcinoma tumors that had both increased *APOBEC3B* expression and the loss of FHIT protein expression [7, 9, 32].

Whereas many studies have focused on the molecular roles of APOBEC3B, and to some extent APOBEC3A, possible cumulative effects of action of APOBEC3A, APOBEC3B, UNG, REV1, and FHIT on generation of APOBEC3B-like mutation motifs and on drug sensitivity in cancer have not been clearly elucidated. To address this question, we investigated the presence of APOBEC3B-like mutational patterns and mRNA expression of the *APOBEC3A*, *APOBEC3B*, *UNG*, *REV1*, and *FHIT* genes in cancer cell lines, in order to identify those cancer cell lines that may have experienced kataegis events. We further examined associations between mutational patterns of APOBEC3 activity, individual cancer types, and chemosensitivity to a variety of antitumor agents. This analysis was carried out using whole-exome sequencing (WES) data, gene expression microarray data, and drug response data for 255 agents from the Cancer Cell Line Encyclopedia (CCLE) [33, 34] and the GDSC resource [30, 35, 36].

## Methods

### Analysis of whole-exome sequencing data

**Fig. 1:**
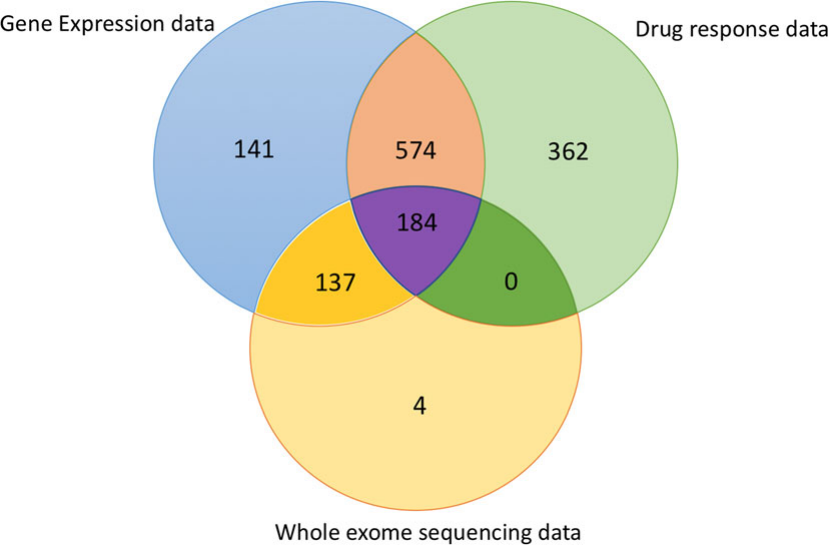
Venn diagram showing the numbers of CCLE cell lines with available data

We downloaded unprocessed WES BAM files, which were available for 325 CCLE cell lines (Fig. 1), from the CCLE project at the National Cancer Institute (NCI) Cancer Genomics Hub; these data are available at the NCI Genomic Data Commons (GDC) data portal [37]. All CCLE WES data had been reported to be sequenced at the Broad Institute using the same version of the Agilent Exome Bait kit, and the same sequencing protocols and data processing pipeline were applied to all samples across all cancer categories [37, 38].

Raw BAM files were preprocessed according to the GATK Best Practices pipeline v. 3.5 as of 15 May 2016 [39–41] using default or recommended parameters for each tool and using Hg19 as the reference human genome assembly. Single nucleotide variant discovery using preprocessed BAM files was carried out with VarScan2 using default parameters [42]. Nucleotide substitutions were filtered by their allele frequencies in the 1000 Genomes Project dataset (August 2015 release), eliminating common population variants with variant allele frequency > 1% in the combined 1000 Genomes Project dataset from all populations [43]. To identify the prevalence of mutation counts, we computed the sum of identified single nucleotide variants across all sequenced exome regions in several separate categories of DNA sequence changes including all SNV mutation counts, as well as C>G, C>T, and C>K counts on one or both genome strands.

We searched the WES nucleotide changes in each cell line for the presence of the three reported APOBEC3B mutation motifs, T(C>K)W, T(C>D)R, or T(C>D)D. This motif representation includes nucleotide IUPAC symbols in three consecutive genome sequence positions, with the two symbols in parenthesis separated by the “>” symbol indicating the direction of nucleotide substitution change. For example, T(C>K)W indicates that the reference genome sequence is 5′-TCA-3′ or 5′-TCT-3′, and an either C>G or C>T substitution was found in the second nucleotide of the triplet. We refer to the three sequence motifs, T(C>K)W, T(C>D)R, and T(C>D)D which were analyzed in this study, as APOBEC-like motifs, in order to distinguish them from the APOBEC mutational signature term, which commonly refers to a matrix of mutational changes that are characteristic of APOBEC activity in the 96-trinucletide format [14, 44]. Both motif and signature formats represent the same patterns of APOBEC mutational activity, and both terms have been used interchangeably in the earlier reports [10].

Because APOBEC activity is characterized by clusters of co-occurring APOBEC motifs with closely spaced mutations on the same genome strands, we further searched each cell line for the presence of kataegis clusters, which were defined using two different but related criteria, either as (a) the same motif occurring on the same genome strand at least five times in a 1000-bp window, to which we refer as 5/1000; or as (b) the same motif occurring on the same genome strand at least six times in a 10,000-bp window, to which we refer as 6/10000. For each cell line, four possible measures of APOBEC-like mutational activity were considered, which defined overall abundance of the APOBEC-like motifs and the abundance and the length of kataegis clusters per WES data of that cell line: (1) the total number of APOBEC-like motifs present in the WES data of each cell line, (2) the number of APOBEC motifs in distinct non-overlapping kataegis regions in WES data of that cell line, (3) the number of distinct non-overlapping kataegis regions in WES data of that cell line, and (4) the total combined length of distinct non-overlapping kataegis regions in WES data of that cell line. We also examined seven overall nucleotide substitution counts for each cell line, including the combined counts of all categories of nucleotide substitutions, and the numbers of C>G, C>T, or C>K substitutions on the reference genome strand and on both genome strands.

### Gene expression analysis

Log_2_-transformed gene expression levels that were available for 1036 cell lines from the Cancer Cell Line Encyclopedia (Fig. 1) were downloaded from the CCLE web resource of the Broad Institute [34]. These measures had been generated using Affymetrix Human Genome U133 Plus 2.0 microarrays and normalized using the Robust Multi-array Average (RMA) algorithm [33, 45]. We analyzed expression of five genes, *APOBEC3B*, *APOBEC3A*, *REV1*, *UNG*, and *FHIT*, which may be involved in generation of *APOBEC*-like mutation motifs. Gene expression data from multiple microarray probes for each gene were averaged. Microarray-derived gene expression values for each gene analyzed in this study were in strong agreement with RNA-seq gene expression measures which recently became available from the CCLE resource [34], with Spearman correlation coefficient *ρ* between 0.883 and 0.947 and the correlation *p* values ≤ 3.33 × 10^−144^ for each of the five genes (data not shown).

To examine possible associations of expression levels of *APOBEC3A* and *APOBEC3B* with the germline *APOBEC3B* gene deletion, we downloaded the copy number status of the *APOBEC3B* gene from the CCLE web resource of the Broad Institute [34]. The copy number data had been generated by the CCLE Consortium using Affymetrix 6.0 SNP arrays, with segmentation of normalized log_2_ ratios of the copy number estimates performed using the circular binary segmentation algorithm [34].

### Analysis of drug response

The IC50 measures of cell line chemosensitivity, representing the total drug inhibitor concentration that reduced cell activity by 50%, were available for 24 drug agents from the Cancer Cell Line Encyclopedia [33] (Fig. 1). These data were downloaded from the CCLE web resource of the Broad Institute [34]. In addition, chemosensitivity values for 251 drug agents for the same cell lines were available from the Genomics of Drug Sensitivity in Cancer resource [30, 35, 36]. GDSC drug response data, in the ln(IC50) format, were obtained from the supplementary Table 4A of Iorio et al. [30]. All drug sensitivity values derived from the CCLE and GDSC datasets were transformed to the log_10_(IC50) scale, to which we further refer as log(IC50). Identities of cell lines present in both CCLE and GDSC datasets were verified using information from Cellosaurus [46]. Drug sensitivity measures for 11 agents which were present in both CCLE and GDSC datasets were analyzed separately for the CCLE and GDSC response measures. For those agents that had duplicate measurements within the GDSC dataset [30], we analyzed their drug response by using a combined average of their drug response measurements from separate experiments. The resulting dataset had 275 CCLE and GDSC drug response measures for 255 distinct antitumor agents. The concordance of drug response measures between the CCLE and GDSC datasets has been studied extensively [47, 48] and validated in an independent screening study [49]. While some authors questioned the extent of the agreement between the two sets of measures [48], most studies confirmed that for the majority of the agents, a solid overall agreement was found between the drug response measures, cell line classification as sensitive or resistant, and molecular predictors of drug sensitivity derived from the GDSC and CCLE datasets [47, 49].

### Statistical analysis

We examined Spearman rank-order correlation among gene expression values, mutation counts, measures of abundance of motifs and kataegis clusters, and drug sensitivity values (log_10_(IC50)) in a combined analysis of all cancer types and within individual types of cancer. The *p* values were adjusted for multiple testing using the Benjamini and Hochberg method of adjustment for false discovery rate, or FDR [50], accounting for 275 drug sensitivity measures, 3 APOBEC-like motifs, 7 different categories of mutation counts, and expression levels of 5 candidate genes. Correlations with FDR adjusted *p* < 0.05 were considered statistically significant. In this report, *ρ* denotes the Spearman correlation coefficient, *p* is a *p* value prior to FDR adjustment, *p*_adj_ is an FDR-adjusted *p* value, *N*_tests_ is the number of correlation tests for which the FDR adjustment of *p* values was made, and *n* is the sample size (the number of cell lines used in estimation or the number of pairs included in the correlation analysis)*.* We focused our discussion on statistically significant moderate or strong correlation results with *p*_adj_ < 0.05 and the absolute value of Spearman correlation coefficient |*ρ*| > 0.25.

Analyses of candidate gene expression levels, motif and kataegis cluster abundance, and correlation analyses were performed both in a combined dataset of all cell lines from different cancer types (pan-cancer analysis), and also within 32 individual cancer categories (Table 1). Many cancer categories were based on TCGA definitions. However, some cancer types from the same organ were grouped in broader categories in order to allow for an inclusion of a broader range of the cell lines than those defined by the TCGA enrollment criteria, and additional categories were included with several cancer types not presented in TCGA (e.g., small cell lung cancer and pediatric tumor categories). These categories are described in Table 1 and in the list of abbreviations. Only those cancer types for which at least 5 cell lines had pairs of available matching data (e.g., WES and expression, expression and drug response, or WES and drug response information) were included in the stratified correlation analyses of individual cancer categories. Accordingly, adjustment for false discovery rate in correlation analyses accounted for 23 cancer categories with ≥ 5 cell lines per category for gene expression comparisons, 17 cancer categories with ≥ 5 cell lines that had both expression and WES data, 26 cancer histologies with expression and chemosensitivity data, and 26 cancer types with ≥ 5 cell lines that had both drug sensitivity data and counts of specific APOBEC-like motif counts derived from WES data. All cell lines with available data were included in the pan-cancer correlation analysis combining all cancer categories. To examine the possible effect of the estrogen receptor status on drug sensitivity of breast cancer cell lines, we performed an additional stratified analysis of ER^+^ and ER^−^ breast cancer cell lines, with their estrogen receptor status defined based on available literature reports [51–54].

**Table 1:** Expression of the five candidate genes in cell lines from different cancer types

Cancer type	*n*	*APOBEC3A*		*APOBEC3B*		*UNG*		*REV1*		*FHIT*	
		Range	Mean ± SD	Range	Mean ± SD	Range	Mean ± SD	Range	Mean ± SD	Range	Mean ± SD
ALL	2	3.40–3.75	3.58 ± 0.25	3.72–9.33	6.53 ± 3.97	10.04–10.56	10.3 ± 0.37	7.52–7.56	7.54 ± 0.03	4.68–7.33	6.01 ± 1.87
BLADDER	27	3.49–6.87	4.11 ± 0.74	3.60–11.51	9.59 ± 1.64	7.86–10.93	9.7 ± 0.81	5.86–8.21	7.01 ± 0.50	4.27–7.10	5.14 ± 0.70
BREAST	59	3.11–6.18	3.88 ± 0.47	3.13–11.28	8.78 ± 2.07	7.00–11.16	9.62 ± 0.82	5.89–8.31	6.98 ± 0.40	4.29–7.26	5.66 ± 0.82
CESC	22	3.14–4.81	3.79 ± 0.33	3.29–10.98	8.87 ± 2.15	8.28–10.39	9.62 ± 0.63	6.45–8.1	7.09 ± 0.45	4.29–7.73	5.87 ± 1.1
CLLE	78	3.29–6.63	3.87 ± 0.46	3.06–11.46	8.01 ± 2.39	7.37–11.50	9.7 ± 0.72	6.53–8.18	7.4 ± 0.36	4.46–10.24	6.59 ± 1.51
COAD/READ	62	3.22–4.54	3.81 ± 0.33	3.02–11.91	8.70 ± 2.30	7.67–10.97	9.75 ± 0.69	6.23–7.76	7.07 ± 0.29	4.30–7.81	6.05 ± 0.89
DA	2	3.36–3.37	3.36 ± 0.01	3.41–3.56	3.49 ± 0.11	9.36–9.95	9.66 ± 0.42	6.97–7.19	7.08 ± 0.16	4.75–4.96	4.85 ± 0.15
EC	26	3.34–5.21	3.90 ± 0.46	3.03–11.81	8.80 ± 2.39	8.51–10.64	9.69 ± 0.55	6.19–7.79	7.06 ± 0.38	4.49–8.34	5.29 ± 0.90
GLIOMA	79	3.27–4.44	3.76 ± 0.25	3.07–11.45	8.42 ± 2.71	7.82–11.09	9.34 ± 0.64	6.32–8.03	7.00 ± 0.34	4.08–7.33	5.13 ± 0.63
HNSC	33	3.34–11.29	4.93 ± 1.86	6.51–11.66	9.54 ± 1.27	7.82–10.35	9.01 ± 0.68	6.25–8.18	7.24 ± 0.45	4.26–6.06	4.85 ± 0.34
LAML	5	3.53–4.74	3.96 ± 0.47	8.14–10.67	9.44 ± 1.06	8.35–10.43	9.67 ± 0.82	7.22–7.69	7.47 ± 0.21	5.80–7.53	6.67 ± 0.80
LCML	1	6.20	6.20	12.56	12.56	9.79	9.79	6.54	6.54	7.69	7.69
LIHC	34	3.34–4.64	3.82 ± 0.3	3.34–12.42	8.39 ± 2.62	7.82–10.72	9.55 ± 0.67	6.07–8.02	6.88 ± 0.39	4.26–7.48	5.33 ± 0.74
MATBCL	60	3.36–5.15	3.84 ± 0.34	3.31–11.76	7.09 ± 2.68	5.81–10.69	9.33 ± 1.1	6.45–8.05	7.27 ± 0.44	4.34–10.67	6.35 ± 1.38
MB	2	3.48–3.83	3.65 ± 0.25	3.48–5.86	4.67 ± 1.68	7.69–9.75	8.72 ± 1.45	6.77–6.94	6.85 ± 0.12	6.00–7.68	6.84 ± 1.19
MEL	59	3.45–4.35	3.87 ± 0.21	3.47–11.81	9.81 ± 1.52	7.32–10.51	9.12 ± 0.62	6.41–7.91	6.91 ± 0.3	4.34–7.67	5.55 ± 0.78
MEN	3	3.65–4.05	3.85 ± 0.20	8.78–9.64	9.08 ± 0.48	8.69–9.72	9.15 ± 0.53	6.47–6.93	6.76 ± 0.25	4.84–5.96	5.25 ± 0.62
MESO	2	3.80–3.95	3.88 ± 0.11	9.92–11.05	10.48 ± 0.79	9.32–9.58	9.45 ± 0.19	6.61–6.8	6.71 ± 0.14	4.18–5.80	4.99 ± 1.15
MGCT	3	3.37–3.62	3.53 ± 0.14	7.19–9.23	8.28 ± 1.03	7.33–8.09	7.77 ± 0.40	6.40–6.73	6.60 ± 0.17	4.73–5.86	5.27 ± 0.57
MM	28	3.46–5.61	4.12 ± 0.48	2.96–12.09	9.52 ± 2.54	7.10–10.85	9.42 ± 0.91	5.83–7.31	6.68 ± 0.36	4.99–8.74	6.96 ± 1.00
NSCLC	186	3.06–7.82	3.79 ± 0.52	3.04–11.92	7.98 ± 2.59	7.85–11.31	9.67 ± 0.64	5.98–8.28	7.08 ± 0.45	4.14–8.11	5.43 ± 0.80
OVARIAN	51	3.31–4.46	3.72 ± 0.24	3.09–10.98	8.06 ± 2.3	7.32–10.71	9.35 ± 0.72	6.29–7.99	7.00 ± 0.32	4.26–8.38	5.79 ± 0.97
PAAD	44	3.28–6.13	3.89 ± 0.50	3.10–11.66	8.95 ± 2.31	7.50–10.95	9.54 ± 0.8	6.48–8.42	7.19 ± 0.36	4.43–7.44	5.34 ± 0.72
PNET	3	3.21–3.59	3.45 ± 0.21	2.94–3.50	3.21 ± 0.28	9.16–10.09	9.54 ± 0.49	6.57–7.58	7.06 ± 0.51	4.43–6.97	5.58 ± 1.29
PRAD	7	3.58–4.07	3.81 ± 0.19	3.33–9.99	8.10 ± 2.18	9.49–11.20	10.15 ± 0.67	6.48–7.65	6.98 ± 0.43	5.04–7.38	5.94 ± 1.01
RCC	36	3.23–4.17	3.70 ± 0.22	3.17–11.25	8.87 ± 1.97	7.70–9.98	9.18 ± 0.51	6.52–7.59	6.96 ± 0.25	4.57–7.05	5.7 ± 0.73
SAR	43	3.38–4.29	3.74 ± 0.23	3.15–11.24	8.37 ± 2.37	7.57–10.73	9.26 ± 0.79	6.48–7.95	7.03 ± 0.39	4.14–6.36	4.87 ± 0.49
SCLC	7	3.01–4.11	3.67 ± 0.39	3.28–11.28	7.38 ± 3.16	9.54–10.60	10.10 ± 0.45	6.88–8.02	7.49 ± 0.41	5.07–6.6	5.71 ± 0.55
STAD	38	3.16–4.58	3.72 ± 0.27	3.21–11.68	7.88 ± 2.68	8.51–10.38	9.53 ± 0.52	5.97–7.75	7.01 ± 0.44	4.35–7.81	5.62 ± 0.9
THCA	13	3.37–4.28	3.66 ± 0.22	3.87–10.97	8.57 ± 2.14	8.07–10.31	9.21 ± 0.62	6.32–7.67	6.94 ± 0.36	4.34–7.63	5.49 ± 0.98
UCEC	6	3.40–4.05	3.66 ± 0.26	3.81–10.99	8.84 ± 2.63	9.36–10.14	9.82 ± 0.32	6.18–7.28	6.78 ± 0.45	4.53–8.11	5.82 ± 1.32
MISC	15	3.36–6.68	4.01 ± 0.80	4.50–11.28	8.83 ± 1.80	7.18–10.42	9.11 ± 1.07	6.47–8.38	7.28 ± 0.53	4.17–6.90	5.26 ± 0.74
*Pan-cancer*	1036	3.23–8.48	3.89 ± 0.61	3.02–12.42	8.43 ± 2.43	7.00–11.50	9.41 ± 0.78	5.83–8.18	7.05 ± 0.42	4.14–10.67	5.74 ± 1.16

*n* number of cell lines for each cancer type with available Affymetrix U133 2.0 plus microarray expression data, *SD* standard deviation, *ALL* acute lymphocytic leukemia, *BLADDER* bladder cancer, *BREAST* breast cancer, *CESC* cervical squamous cell carcinoma and endocervical adenocarcinoma, *CLLE* chronic lymphocytic leukemia, *COAD/READ* colon adenocarcinoma and rectum adenocarcinoma, *DA* duodenal adenocarcinoma, *EC* esophageal cancer, *GLIOMA* glioma brain tumors, *HNSC* head and neck squamous cell carcinoma, *LAML* acute myeloid leukemia, *LCML* chronic myelogenous leukemia, *LIHC* liver hepatocellular carcinoma, *MATBCL* mature B cell lymphoma, *MB* medulloblastoma, *MEL* melanoma, *MEN* meningioma, *MESO* mesothelioma, *MGCT* malignant giant cell tumor of bone, *MM* multiple myeloma, *NSCLC* non-small cell lung cancer, *OVARIAN* ovarian cancer, *PAAD* pancreatic adenocarcinoma, *PNET* primitive neuroectodermal tumors, *PRAD* prostate adenocarcinoma, *RCC* renal cell carcinoma, *SAR* sarcoma, *SCLC* small cell lung cancer, *STAD* stomach adenocarcinoma, *THCA* thyroid carcinoma, *UCEC* uterine corpus endometrial carcinoma, *MISC* other miscellaneous categories of cancer including rare cancers or cancers with unspecified information, *Pan-cancer* combined analysis of all cancer categories

Bioinformatic and statistical analyses were performed using Python v. 2.7 and R v. 3.4.

## Results

### Candidate gene expression patterns

**Fig. 2:**
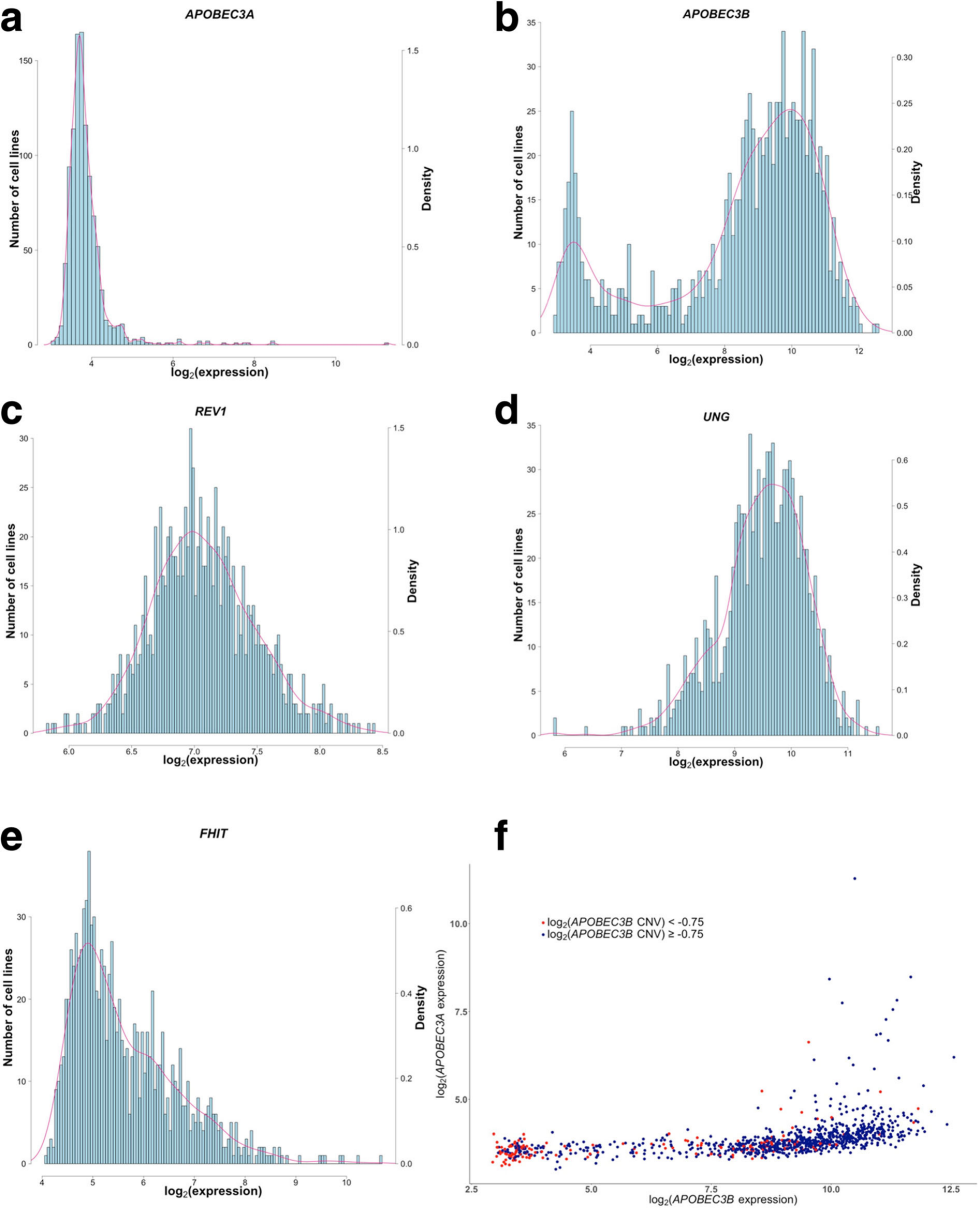
**a**–**e** Histograms and density functions showing the distributions of expression of the five candidate genes in the cell lines. **a** *APOBEC3A*. **b** *APOBEC3B*. **c** *REV1*. **d** *UNG*. **e** *FHIT*. Horizontal scale represents log_2_-transformed gene expression values. The left vertical scale represents cell line counts, whereas the right vertical scale represents density values. **f** A scatterplot of *APOBEC3B* vs *APOBEC3A* expression in 1012 cell lines from the CCLE microarray expression dataset which shows the copy number status of the *APOBEC3B* gene according to the CCLE data [33]. Cell lines with log_2_(normalized ratio of *APOBEC3B* copy number estimate) ≥ − 0.75 are shown in blue, whereas those with log_2_(normalized ratio of *APOBEC3B* copy number estimate) < − 0.75 are shown in red

Table 1 provides expression levels of each candidate gene in the cell lines from individual cancer types as well as average gene expression levels in the pan-cancer dataset. Examination of gene expression measures in the pan-cancer dataset showed a bimodal distribution of *APOBEC3B* expression (Fig. 2b), whereas *APOBEC3A*, *REV1*, *UNG*, and *FHIT* had unimodal distributions of their expression measures (Fig. 2a, c–e). Analysis of the *APOBEC3B* copy number status showed that low levels of *APOBEC3B* expression were observed both in the samples with the *APOBEC3B* gene loss due to the *APOBEC3B* germline deletion polymorphism and in a number of samples without the loss of the *APOBEC3B* gene (Fig. 2f). The expression of *APOBEC3A* was low in many of the cell lines (mean = 3.89; Table 1; Fig. 2a), in agreement with an earlier study [7].

When compared to the mean *APOBEC3A* and *APOBEC3B* gene expression levels in the pan-cancer dataset (Table 1; mean expression values of 3.89 and 8.43, respectively), cell lines from the following cancer categories had elevated expression values of both *APOBEC3A* and *APOBEC3B*: bladder (mean values of 4.11 and 9.59, respectively), head and neck (HNSC; 4.93 and 9.54), chronic myelogenous leukemia (LCML; 6.20 and 12.56), and multiple myeloma (MM; 4.12 and 9.52). Several other cancer types had increased levels of expression of the *APOBEC3B* gene, but their mean expression levels of *APOBEC3A* were comparable to the mean *APOBEC3A* expression across all cancer types. Among the cancer categories with ≥ 5 cell lines, these included acute myeloid leukemia (LAML; mean *APOBEC3B* expression of 9.44) and melanoma (MEL; 9.81).

Our findings of elevated *APOBEC3B* and *APOBEC3A* expression in cell lines from several cancer types presented in Table 1 were consistent with earlier studies of patient-based samples. Many earlier studies reported elevated expression and activity of *APOBEC3B* and *APOBEC3A* in bladder cancer and of *APOBEC3B* in head and neck cancer patients [5, 6, 9, 55, 56]. APOBEC-derived mutagenesis is considered to be the predominant mutation source in 65% of invasive bladder cancers in the TCGA dataset [57]. Similarly, a genomic signature attributed to APOBEC3 activity was reported in a subset of patients with all melanoma subtypes, although C>T transitions attributed to APOBEC activity could be confounded with UV-induced substitutions in many melanoma cells [12, 57, 58]. Increased expression and activity of both *APOBEC3A* and *APOBEC3B* were also reported in multiple myeloma patients, most commonly in those with the t(14:16) translocation, which was associated with poor survival [56, 59, 60].

Elevated levels of *UNG* expression, but not of other candidate genes, were found in the prostate adenocarcinoma (PRAD; 10.15) and small cell lung cancer (SCLC; 10.10) cell lines (Table 1). Clusters of single-strand mutation patterns suggestive of APOBEC activity were previously reported in prostate cancer [56], and it may be possible that increased *UNG* expression may contribute to mutagenesis in that cancer category. Because abrogated FHIT activity may increase the levels of mutagenesis both as a standalone mechanism and synergistically with APOBEC3B [7, 9, 32], we note that cell lines from several cancer types including head and neck (4.85) and sarcoma (4.87) had a considerably lower mean *FHIT* expression than the pan-cancer average (5.74). Therefore, both high levels of *APOBEC3B* and *APOBEC3A* and low levels of *FHIT* expression may influence APOBEC mutagenesis in the head and neck cancer.

Expression levels of *APOBEC3B* showed strong and statistically significant positive correlation with *APOBEC3A* expression in 21 cancer categories (Table 2; *ρ* between 0.576 and 1.000; *p*_adj_ < 0.05). These categories (NSCLC, LAML, GLIOMA, COAD/READ, MATBCL, STAD, OVARIAN, RCC, MEL, CLLE, SAR, BREAST, BLADDER, LIHC, EC, PAAD, HNSC, CESC, MM, THCA, and UCEC; see legend of Table 1 and the list of abbreviations for their description) included both solid tumors and hematological malignancies. A strong positive and highly significant correlation between *APOBEC3B* and *APOBEC3A* expression was also observed in the pan-cancer analysis (Table 2; *ρ* = 0.714, *p*_adj_ < 0.001, *n* = 1036, *N*_tests_ = 10). Interestingly, breast cancer cell lines were among the cancer types with positive correlation between *APOBEC3A* and *APOBEC3B* expression (Table 2). Earlier studies found strong evidence for increased *APOBEC3B* activity and mutagenesis in a subset of breast cancers [7, 20, 21, 61] and with APOBEC signature enrichment in the HER2 breast cancer subtype and in triple negative breast cancer (TNBC) [6, 62]; however, a study of breast cancer cell lines found generally low levels of *APOBEC3A* expression [29]. Possible molecular impact of coordinated expression levels of *APOBEC3A* and *APOBEC3B* in the breast cancer cell lines analyzed in our study is of interest and requires further investigation.

**Table 2:** Significant correlations among candidate gene expression levels

Gene 1	Gene 2	*n*	*ρ*	*p*	*p*_adj_	Cancer category
Within individual cancer categories						
*APOBEC3B*	*APOBEC3A*	186	0.741	1.15 × 10^−33^	2.64 × 10^−31^	NSCLC
*APOBEC3B*	*APOBEC3A*	5	1.000	1.40 × 10^−24^	1.61 × 10^−22^	LAML
*APOBEC3B*	*APOBEC3A*	62	0.759	8.88 × 10^−13^	6.81 × 10^−11^	COAD/READ
*APOBEC3B*	*APOBEC3A*	78	0.690	2.83 × 10^−12^	1.44 × 10^−10^	CLLE
*APOBEC3B*	*APOBEC3A*	79	0.686	3.12 × 10^−12^	1.44 × 10^−10^	GLIOMA
*APOBEC3B*	*APOBEC3A*	38	0.811	6.60 × 10^−10^	2.53 × 10^−8^	STAD
*APOBEC3B*	*APOBEC3A*	51	0.712	4.82 × 10^−9^	1.58 × 10^−7^	OVARIAN
*APOBEC3B*	*APOBEC3A*	44	0.746	6.12 × 10^−9^	1.76 × 10^−7^	PAAD
*APOBEC3B*	*APOBEC3A*	60	0.651	1.73 × 10^−8^	4.43 × 10^−7^	MATBCL
*APOBEC3B*	*APOBEC3A*	59	0.612	2.65 × 10^−7^	6.09 × 10^−6^	BREAST
*APOBEC3B*	*APOBEC3A*	26	0.805	7.04 × 10^−7^	1.47 × 10^−5^	EC
*UNG*	*REV1*	186	0.344	1.56 × 10^−6^	2.98 × 10^−5^	NSCLC
*APOBEC3B*	*APOBEC3A*	59	0.576	1.81 × 10^−6^	3.20 × 10^−5^	MEL
*APOBEC3B*	*APOBEC3A*	27	0.773	2.30 × 10^−6^	3.78 × 10^−5^	BLADDER
*APOBEC3B*	*APOBEC3A*	43	0.639	3.95 × 10^−6^	6.06 × 10^−5^	SAR
*APOBEC3B*	*APOBEC3A*	36	0.637	3.00 × 10^−5^	0.0004	RCC
*APOBEC3B*	*APOBEC3A*	34	0.645	3.83 × 10^−5^	0.0005	LIHC
*APOBEC3B*	*APOBEC3A*	22	0.747	6.48 × 10^−5^	0.0008	CESC
*APOBEC3B*	*APOBEC3A*	33	0.636	6.87 × 10^−5^	0.0008	HNSC
*APOBEC3B*	*FHIT*	79	− 0.407	0.0002	0.0022	GLIOMA
*APOBEC3B*	*APOBEC3A*	28	0.632	0.0003	0.0034	MM
*APOBEC3B*	*APOBEC3A*	13	0.769	0.0021	0.0221	THCA
*APOBEC3B*	*UNG*	60	− 0.372	0.0034	0.0342	MATBCL
*APOBEC3A*	*UNG*	78	− 0.324	0.0039	0.0369	CLLE
*APOBEC3B*	*APOBEC3A*	6	0.943	0.0048	0.0442	UCEC
Across all cancer categories						
*APOBEC3B*	*APOBEC3A*	1036	0.714	1.91 × 10^−162^	1.91 × 10^−161^	Pan-cancer
*UNG*	*REV1*	1036	0.189	8.04 × 10^−10^	4.02 × 10^−9^	Pan-cancer
*APOBEC3B*	*REV1*	1036	− 0.118	0.0001	0.0005	Pan-cancer
*APOBEC3B*	*FHIT*	1036	− 0.088	0.0046	0.0115	Pan-cancer
*APOBEC3B*	*UNG*	1036	− 0.070	0.0251	0.0426	Pan-cancer
*APOBEC3A*	*UNG*	1036	− 0.068	0.0291	0.0426	Pan-cancer
*UNG*	*FHIT*	1036	0.068	0.0298	0.0426	Pan-cancer

Listed are significant correlations with *p*_adj_ < 0.05. The *p* values were adjusted for false discovery rate accounting for five genes (*N*_tests_ = 10). Among individual cancer categories, FDR adjustment also accounted for 23 cancer categories with ≥ 5 cell lines with available expression data in both genes (*N*_tests_ = 230). Abbreviations of cancer categories are provided in the legend of Table 1

*n* sample size for correlation analysis, *ρ* Spearman correlation coefficient, *p p* value prior to FDR adjustment, *p*_*adj*_ FDR-adjusted *p* value

Expression of *APOBEC3B* was significantly negatively correlated with *FHIT* expression in glioma cell lines (*ρ* = − 0.407, *p*_adj_ = 0.0022, *n* = 79, *N*_tests_ = 230). This negative correlation is notable because low levels of the *FHIT* gene expression or the loss of FHIT function have been reported to have a cooperative effect with APOBEC3B in mutagenesis, even though *APOBEC3B* overexpression and DNA damage induced by the replication stress caused by the loss of FHIT have been proposed to occur independently from each other [7, 9, 32]. Negative correlation between *APOBEC3B* and *FHIT* expression levels could potentially produce hypermutated clusters in those cells where *APOBEC3B* expression were elevated and *FHIT* expression were diminished. However, this did not appear to be the case because in our analysis of glioma cell lines, which included astrocytoma, lower-grade glioma, and glioblastoma multiforme cell lines, mean APOBEC3B and *FHIT* expression levels were comparable to those in the pan-cancer dataset (Table 1). Such expression levels were consistent with earlier studies [12, 63], which had reported low levels of *APOBEC3B* in lower-grade glioma TCGA patient samples and had suggested that mutation processes in glioma tumors could be caused by mechanisms other than APOBEC mutagenesis.

*UNG* expression was negatively correlated with *APOBEC3B* expression in mature B cell lymphoma cell lines (MATBCL; *ρ* = − 0.372, *p*_adj_ = 0.034, *n* = 60, *N*_tests_ = 230) and with *APOBEC3A* expression in chronic lymphocytic leukemia cells (CLLE; *ρ* = − 0.324, *p*_adj_ = 0.037, *n* = 78, *N*_tests_ = 230). Expression levels of *UNG* and *REV1* were significantly positively correlated in non-small cell lung cancer cell lines (NSCLC; *ρ* = 0.344, *p*_adj_ *=* 2.98 × 10^−5^, *n* = 186, *N*_tests_ = 230).

### APOBEC-like mutation motifs and mutation loads in cancer cell lines

**Fig. 3:**
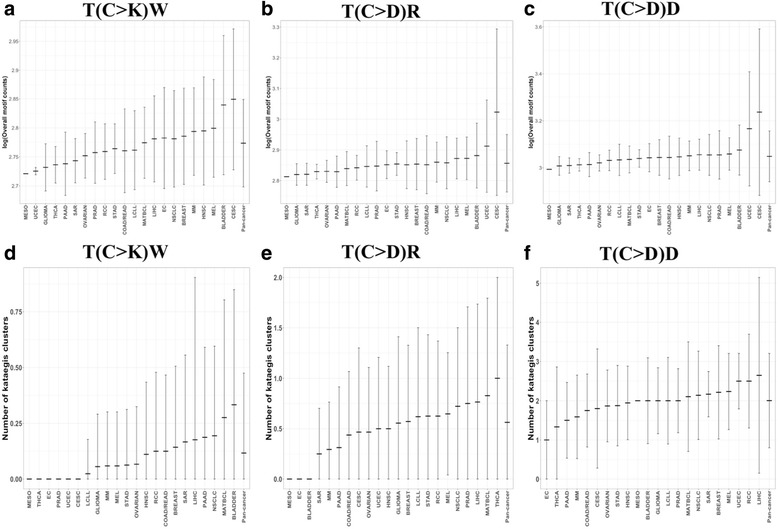
**a**–**c** Overall motif counts in different cancer types and across all cell lines (pan-cancer analysis). The *y* axis is presented on the log_10_ scale. **a** T(C>K)W motif counts. **b** T(C>D)R motif counts. **c** T(C>D)D motif counts. **d**–**f** Numbers of distinct, not overlapping 5/1000 kataegis clusters with ≥ 5 motifs on the same genome strand per 1000 bp in different cancer types and in the pan-cancer dataset. **d** T(C>K)W motif counts. **e** T(C>D)R motif counts. **f** T(C>D)D motif counts. Horizontal middle bars show the mean for each cancer category. Vertical bars show mean ± standard deviation. Negative values of (mean − standard deviation) in **d** and **e** were truncated at 0. Cancer categories with no vertical columns had no predicted kataegis clusters (**d**–**f**) and/or too few cell lines to compute the standard deviation (*n* = 2 for mesothelioma, **a**–**c**)

Prevalence of mutation counts and single nucleotide positions in the combined analysis of all cancer categories and within individual cancer types in the 325 cell lines with available WES data is provided in Table 3. Because some individual cancer categories had small sample sizes of the cell lines with WES data, not all mutation counts in cell lines were representative of mutation counts in large patient samples for specific cancer types. For example, mutation counts at single nucleotide positions in the bladder cancer category, which included six cell lines, were lower than the typically high mutation rates that are commonly seen in bladder cancer patients [12, 57, 64]. However, clusters of mutations in genome regions have been reported to provide a more robust representation of mutational processes in tumor genomes that do average mutation rates at single positions [13]. As discussed below, the prevalence of APOBEC-like motifs and kataegis clusters (Fig. 3) in bladder cancer cell lines and in cell lines from several other cancer categories of our dataset was generally consistent with the relative ranking of cancer categories previously described using patient data.

**Table 3:** Prevalence of mutation counts in the whole-exome sequencing data

		C>G		C>T		C>K		All SNV counts	
Cancer type	*n*	Range	Mean ± SD	Range	Mean ± SD	Range	Mean ± SD	Range	Mean ± SD
BLADDER	6	5297–7790	6564 ± 968	19,570–28,590	24,077 ± 3408	24,867–36,380	30,640 ± 4373	54,376–78,947	66,878 ± 9294
BREAST	14	6141–8460	6860 ± 566	22,789–33,262	25,705 ± 2548	28,930–41,722	32,564 ± 3102	63,719–89,515	71,153 ± 6292
CESC	15	6309–7994	7095 ± 530	23,581–34,126	28,889 ± 3587	29,890–41,452	35,984 ± 3926	65,633–101,318	79,411 ± 9753
COAD/READ	16	5332–7595	6657 ± 664	20,925–36,866	25,962 ± 3970	26,257–44,461	32,620 ± 4555	59,552–89,617	70,796 ± 8093
EC	3	6703–7357	6954 ± 353	24,685–27,760	25,752 ± 1740	31,486–35,117	32,706 ± 2088	68,907–76,008	71,472 ± 3940
GLIOMA	18	5833–7682	6713 ± 458	21,183–28,151	24,924 ± 1644	27,016–35,833	31,637 ± 2096	60,001–78,102	69,420 ± 4414
HNSC	18	5195–7378	6714 ± 467	20,073–27,050	25,054 ± 1618	25,268–34,428	31,768 ± 2074	55,628–75,813	69,801 ± 4531
CLLE	42	4235–8400	6974 ± 723	17,410–32,021	26,545 ± 2888	21,645–40,010	33,520 ± 3549	47,685–86,517	72,972 ± 7267
LIHC	17	5864–8444	7007 ± 497	22,051–30,565	25,793 ± 1781	27,915–39,009	32,800 ± 2266	61,208–85,224	72,102 ± 4850
MATBCL	29	6350–8912	7209 ± 593	23,674–33,141	27,029 ± 2170	30,125–42,053	34,239 ± 2751	66,014–91,667	74,874 ± 5935
MEL	17	5722–8448	6759 ± 631	22,174–31,819	25,874 ± 2324	27,896–40,267	32,633 ± 2945	60,650–87,815	70,805 ± 6434
MESO	1	6112	6112	21,790	21,790	27,902	27,902	62,016	62,016
MM	17	6187–8662	6840 ± 628	22,773–32,455	25,456 ± 2338	28,960–41,117	32,296 ± 2961	63,335–88,898	70,785 ± 6192
NSCLC	36	5509–8739	6927 ± 768	20,710–32,767	25,641 ± 2666	26,219–41,506	32,567 ± 3424	57,506–90,159	71,563 ± 7520
OVARIAN	15	5951–7461	6682 ± 503	22,453–27,222	25,077 ± 1500	28,433–34,683	31,760 ± 1988	62,699–75,986	69,753 ± 4383
PAAD	16	5011–7432	6640 ± 588	19,327–27,658	24,801 ± 2144	24,338–35,090	31,441 ± 2725	53,010–76,653	68,905 ± 5941
PRAD	4	5699–6889	6423 ± 512	20,538–28,059	25,092 ± 3450	26,237–34,948	31,515 ± 3947	57,717–74,831	68,722 ± 8018
RCC	8	6521–7566	6980 ± 411	24,508–27,801	26,133 ± 1383	31,082–35,264	33,114 ± 1783	68,091–77,777	72,638 ± 4113
SAR	12	6336–7808	6968 ± 423	23,647–29,155	26,129 ± 1610	29,983–36,963	33,098 ± 2027	65,833–81,175	72,342 ± 4357
STAD	16	5861–7530	6807 ± 448	21,971–28,460	25,305 ± 1763	27,832–35,741	32,112 ± 2199	61,311–79,632	70,672 ± 4843
THCA	3	5811–6918	6463 ± 579	22,080–25,849	24,363 ± 2007	27,891–32,767	30,826 ± 2586	61,598–71,836	67,720 ± 5406
UCEC	2	6063–6489	6276 ± 301	24,128–24,223	24,176 ± 67	30,286–30,617	30,452 ± 234	66,406–67,542	66,974 ± 803
Pan-cancer	325	4235–8912	6865 ± 618	17,410–36,866	25,867 ± 2575	21,645–44,461	32,732 ± 3139	47,685–101,318	71,661 ± 6693

Shown are counts of C>T, C>G, and C>K substitutions on both genome strands, and of any types of SNV variants representing nucleotide substitutions

*K* G or T, *SD* standard deviation, *SNV* single nucleotide variant, *n* number of cell lines

Table 4 shows the abundance of the three APOBEC-like motifs and their predicted kataegis clusters in WES sequence data of CCLE cell lines in the combined analysis of all cancer types. Among the three motifs, the commonly reported APOBEC3B motif with narrow specificity, T(C>K)W [7], resulted in the smallest numbers of predicted motifs (mean ± standard deviation of 603.58 ± 121.17) and kataegis clusters (0.12 ± 0.36 clusters of 5 motifs in 1000-bp windows per cell line), followed by higher numbers of motifs (743.51 ± 317.68) and kataegis clusters (0.56 ± 0.77) for the T(C>D)R motif. The highest numbers of APOBEC-like motifs (1184.94 ± 887.46) and clusters (2 ± 1.2 per cell line) were predicted for the least specific motif, T(C>D)D. That motif included possible nucleotide changes of both motifs T(C>K)W and T(C>D)R. Similar patterns were observed for the combined length of the 5/1000 kataegis clusters, the numbers of motifs in distinct 5/1000 clusters, or when considering 6/10000 kataegis clusters (Table 4).

**Table 4:** Prevalence of APOBEC mutation motifs and kataegis clusters in a combined analysis of all cancer categories

Measure	T(C>K)W		T(C>D)R		T(C>D)D	
	Range	Mean ± SD	Range	Mean ± SD	Range	Mean ± SD
Total motif count	381–1369	603.58 ± 121.17	465–4633	743.51 ± 317.68	715–13,461	1184.94 ± 887.46
Predicted non-overlapping kataegis clusters, 5/1000						
Number of motifs in distinct clusters	0–16	0.6 ± 1.87	0–21	2.9 ± 3.99	0–69	10.71 ± 6.8
Number of distinct clusters	0–3	0.12 ± 0.36	0–4	0.56 ± 0.77	0–11	2 ± 1.2
Combined length (bp) of distinct clusters	0–1994	76.95 ± 238.6	0–3148	418.47 ± 615.59	0–7484	1327.31 ± 894.04
Predicted non-overlapping kataegis clusters, 6/10000						
Number of motifs in distinct non-overlapping clusters	0–95	0.87 ± 6.08	0–93	3.93 ± 8.34	0–221	10.26 ± 16.69
Number of distinct non-overlapping clusters	0–10	0.11 ± 0.67	0–8	0.53 ± 0.86	0–18	1.45 ± 1.65
Combined length (bp) of distinct clusters	0–89,163	750.11 ± 5802.03	0–78,974	2997.94 ± 7701	0–147,285	5323.27 ± 12,925.39

Shown are values per cell line, computed using whole-exome sequence data of each cell line

*SD* standard deviation, *5/1000* a kataegis cluster with ≥ 5 motifs on the same genome strand per 1000 bp, *6/10000* a kataegis cluster with ≥ 6 motifs on the same genome strand per 10,000 bp

Similar trends in the abundance of motifs and kataegis-like clusters were also observed among individual cancer categories, as presented in Fig. 3, which shows the distributions of motif counts and numbers of the 5/1000 kataegis clusters among cell lines from different cancer types. For the most specific APOBEC motif, T(C>K)W, the highest mean number of motifs per cell line was observed in cervical squamous cell carcinoma and endocervical adenocarcinoma (CESC; mean = 736 motifs per cell line), followed by bladder cancer (mean = 716 motifs), and melanoma (mean = 642 motifs; Fig. 3a). These categories have been reported to have high levels of APOBEC3 activity [12], although some C>K mutations in melanoma were likely caused by ultraviolet (UV) radiation [10, 14]. The highest mean number of the 5/1000 kataegis clusters with the T(C>K)W motif was observed in bladder cancer (mean = 0.33 clusters per cell line), followed by mature B cell lymphoma (MATBCL; mean = 0.28 clusters), and NSCLC (mean = 0.19 clusters; Fig. 3d). For a less specific motif, T(C>D)R, the three cell line categories with the highest mean numbers of motifs were CESC (mean = 1343 motifs per cell line), uterine corpus endometrial carcinoma (UCEC; mean = 842 motifs), and bladder cancer (mean = 781 motifs; Fig. 3b). While high levels of APOBEC3 activity have been reported in these cancers, additional mechanisms may also be contributing to UCEC mutagenesis [12]; in addition, only two UCEC cell lines had WES data, resulting in a very small sample size. The highest mean number of the 5/1000 kataegis clusters with the T(C>D)R motif was observed for THCA (mean = 1.00 cluster), followed by MATBCL (mean = 0.83 clusters) and the liver hepatocellular carcinoma (LIHC; mean = 0.76; Fig. 3e). The highest counts of the third and the least specific motif, T(C>D)D, were found in CESC (mean = 2744 motifs per cell line), UCEC (mean = 2177 motifs), and bladder cancer cell lines (mean = 1221 motifs; Fig. 3c). These cancer categories been reported to have strong APOBEC3 activity [12]. The highest numbers of 5/1000 kataegis clusters with the T(C>D)D motif were observed in LIHC (mean = 2.65 clusters), renal cell carcinoma (RCC; mean = 2.50 clusters), and UCEC (mean = 2.50 clusters; Fig. 3f). When 6/10000 kataegis clusters (data not shown), the two cancer types with the highest mean numbers of kataegis clusters were LIHC (mean = 0.76 clusters for T(C>K)W, 1.24 clusters for T(C>D)R, and 3.24 clusters for the T(C>D)D motif) and RCC (mean = 0.38, 0.88, and 2.13 clusters, respectively).

Our findings for bladder cancer, melanoma, non-small cell lung cancer, uterine corpus endometrial carcinoma, and prostate adenocarcinoma were consistent with previous reports which suggested the roles for APOBEC3 mutagenesis in those cancer types [5, 6, 12, 57, 60, 65]. In contrast, APOBEC3B was reported to be less likely to play a role in mutagenesis of renal cell carcinoma cell lines [6, 12, 65], suggesting that high prevalence of mutation clusters in the RCC cell lines observed in our study could be generated by molecular factors other than APOBEC3B. The increased prevalence of mutagenic clusters in mature B cell lymphoma cell lines may be explained by the effects of translesion synthesis DNA polymerase η [13, 66]. It is also possible that some of the mutations in MATBCL could be explained by a partial overlap of the motifs examined in our study with a characteristic signature for another member of the APOBEC family, the activation-induced cytidine deaminase (AID), which has been linked to mutagenesis in MATBCL. However, AID has a distinct preference for the WRCY/RGYW motif, and its mutational signature is distinguishable from that of APOBEC3A/B [9, 10, 16, 67], and therefore, it is less likely that an increased number of APOBEC3-like motifs found in MATBCL could be attributed to AID activity.

The statistically significantly increased *APOBEC3B* gene and protein expression in hepatocellular carcinoma as compared to non-tumor tissues, as well as the high rates of C>D mutation changes in the genomes of hepatocellular carcinoma tumors have been documented previously [68–72], in agreement with an increased prevalence of APOBEC-like motifs in LIHC cell lines in our dataset (Fig. 3). However, the potential role of APOBEC3B in mutagenesis in hepatocellular carcinoma has been controversial, with some studies reporting its tumor-inducing roles and others suggesting that it may play a role in tumor suppression. Mutation signature analysis found the presence of signatures other than those induced by APOBEC3B in patient samples of hepatocellular carcinoma [11]. Other molecular factors such as transcription-coupled repair, inhibition of UNG accompanied by APOBEC3G-induced hypermutation, translesion synthesis by one of the DNA polymerases, or the role of APOBEC1 have been implicated in mutagenesis of hepatocellular carcinomas [10, 17, 69–71, 73, 74], and therefore it may be possible that the increased prevalence of APOBEC-like motif clusters in LIHC cell lines may be caused by factors other than APOBEC3B.

### Correlation of gene expression levels with mutation counts and with prevalence of APOBEC-like motifs

Analysis of the pan-cancer dataset showed a very weak correlation (|*r*| ≤ 0.161) of expression levels of candidate genes with motif counts, counts of kataegis clusters, and mutation counts in the WES data. None of these correlations were statistically significant (*p*_adj_ ≥ 0.08). Among the five candidate genes, the strongest correlations were observed for *APOBEC3A*, *APOBEC3B*, and *REV1.*

Among individual cancer types, we observed a strong (*ρ* between − 0.738 and − 0.902) and statistically significant (*p*_adj_ < 0.05) negative correlation of the frequencies of C>T, C>G, and C>K substitutions and overall nucleotide substitution counts with *REV1* expression in sarcoma and *UNG* expression in melanoma (Table 5). The third ranking gene for correlations with mutation counts was *APOBEC3A.* Although it did not reach the stringent threshold of FDR adjusted *p* < 0.05, it showed strong positive correlations (*ρ* ≤ 0.90, *p*_adj_ ≥ 0.07) with several categories of mutation counts in renal cell carcinoma. *APOBEC3B* expression also had the strongest correlation with mutation counts in RCC as opposed to other cancer categories; however, such correlations for *APOBEC3B* were somewhat weaker and less significant (*ρ* ≤ 0.86, *p*_adj_ ≥ 0.16) than those for *APOBEC3A* (data not shown). These correlation results suggest a strong contribution of REV1, UNG, and possibly APOBEC3A to overall mutagenesis in sarcoma, melanoma, and renal cell carcinoma, respectively. A large proportion of C>T and C>G substitutions in melanoma cell lines were likely generated via mutagenic processes related to UV radiation exposure [10, 14]. However, the role for APOBEC3 in melanoma mutagenesis has also been established in a subset of melanomas [58], and experimental evidence has suggested an important role of APOBEC3A generating mutations specific to skin lesions [75].

**Table 5:** Statistically significant correlations of gene expression levels with mutation counts

Gene	Mutation count	*n*	*ρ*	*p*	*p*_adj_	Cancer type
*REV1*	C>K^b^	12	− 0.902	6.00 × 10^−5^	0.0114	Sarcoma
*REV1*	C>K^a^	12	− 0.895	8.37 × 10^−5^	0.0114	Sarcoma
*REV1*	C>T^b^	12	− 0.895	8.37 × 10^−5^	0.0114	Sarcoma
*REV1*	Any	12	− 0.881	0.0002	0.0114	Sarcoma
*REV1*	C>T^a^	12	− 0.881	0.0002	0.0114	Sarcoma
*REV1*	C>G^a^	12	− 0.867	0.0003	0.0119	Sarcoma
*REV1*	C>G^b^	12	− 0.867	0.0003	0.0119	Sarcoma
*UNG*	C>K^a^	17	− 0.816	6.45 × 10^−5^	0.0114	Melanoma
*UNG*	Any	17	− 0.799	0.0001	0.0114	Melanoma
*UNG*	C>K^b^	17	− 0.797	0.0001	0.0114	Melanoma
*UNG*	C>G^a^	17	− 0.787	0.0002	0.0118	Melanoma
*UNG*	C>T^a^	17	− 0.779	0.0002	0.0119	Melanoma
*UNG*	C>T^b^	17	− 0.777	0.0002	0.0119	Melanoma
*UNG*	C>G^b^	17	− 0.738	0.0007	0.0308	Melanoma

Shown are correlations of gene expression levels with overall mutation counts in the WES data with *p*_adj_ < 0.05. These *p* values were FDR adjusted for multiple comparisons that included 5 candidate genes, 17 cancer categories with ≥ 5 cell lines in each category having both WES and expression data, and 7 categories of mutation counts including C>T, C>G, and C>K on one or both genome strands, as well as overall single nucleotide variant counts (*N*_tests_ = 595). “>” indicates the direction of substitution change

*Any* all types of nucleotide substitutions, *K* G or T, *n* sample size for correlation analysis, *ρ* Spearman correlation coefficient, *p p* value prior to FDR adjustment, *p*_*adj*_ FDR-adjusted *p* value

^a^Mutation counts on the reference genome strand only

^b^Mutation counts on both genome strands

Among the correlations of gene expression levels with APOBEC-like motif counts and measures of kataegis, significant or nearly significant correlations were observed for *UNG* expression with kataegis measures (*ρ* between *−* 0.81 and − 0.80, 0.039 ≤ *p*_adj_ ≤ 0.063, *n* = 17, *N*_tests_ = 475) of the T(C>D)D motif in melanoma, and for *APOBEC3A* expression with motif counts and kataegis measures in renal cell carcinoma (*ρ* between 0.93 and 0.98, 0.008 ≤ *p*_adj_ ≤ 0.087 with *n* = 8 and *N*_tests_ = 510 for the T(C>D)R and T(C>D)D motifs; data not shown).

### Correlation of candidate gene expression with chemosensitivity

Table 6 lists the strongest (|*ρ*| > 0.25) statistically significant (*p*_adj_ < 0.05) correlations between candidate gene expression levels and cell line chemosensitivity to drug treatment. Several strong correlations were observed in PAAD, PRAD, CESC, MM, SAR, RCC, NSCLC, MEL, and SCLC cell lines.

**Table 6:** Strongest significant correlations between candidate gene expression and drug sensitivity

Cancer category	Gene	Agent	*n*	*ρ*	*p*	*p*_adj_	Drug action/alternative name	Reference
PAAD	*APOBEC3A*	JQ1^a^	28	− 0.819	9.70 × 10^−8^	0.0001	BET inhibitor	[78]
PRAD	*APOBEC3A*	PD-0332991^a^	5	− 1.000	1.40 × 10^−24^	1.75 × 10^−21^	Palbociclib; CDK 4/6 inhibitor	[100]
PRAD	*APOBEC3B*	GDC0941^a^	5	− 1.000	1.40 × 10^−24^	1.75 × 10^−21^	Pictilisib; pan-class I PI3K inhibitor	[101]
PRAD	*APOBEC3B*	KIN001-260^a^	5	− 1.000	1.40 × 10^−24^	1.75 × 10^−21^	IKKb inhibitor	[36]
PRAD	*APOBEC3B*	EHT 1864^a^	5	− 1.000	1.40 × 10^−24^	1.75 × 10^−21^	Rac inhibitor	[102]
PRAD	*APOBEC3B*	Nutlin-3a^a^	5	− 1.000	1.40 × 10^−24^	1.75 × 10^−21^	Inhibitor of MDM2-p53 interaction	[87]
CESC	*APOBEC3B*	ZM-447439^a^	5	− 1.000	1.40 × 10^−24^	1.75 × 10^−21^	Aurora kinase inhibitor	[103]
MM	*APOBEC3B*	QL-VIII-58^a^	5	− 1.000	1.40 × 10^−24^	1.75 × 10^−21^	Inhibitor of mTOR and ATR signaling	[36]
MM	*APOBEC3B*	ZG-10^a^	5	− 1.000	1.40 × 10^−24^	1.75 × 10^−21^	Inhibitor of JNK1 and p38 signaling	[36]
SAR	*APOBEC3B*	TGX221^a^	6	− 1.000	< 4.95 × 10^−324^	< 4.95 × 10^−324^	PI3Kβ inhibitor	[36]
CESC	*REV1*	MLN4924^a^	5	− 1.000	1.40 × 10^−24^	1.75 × 10^−21^	Pevodenistat; NAE inhibitor	[36]
RCC	*REV1*	XMD8-92^a^	6	− 1.000	< 4.95 × 10^−324^	< 4.95 × 10^−324^	BMK1/ERK5 inhibitor	[104]
NSCLC	*REV1*	RDEA119^a^	123	0.381	1.35 × 10^−5^	0.0153	Refametinib; BAY 86-9766; MEK inhibitor	[83]
NSCLC	*REV1*	PD-0325901^a^	106	0.405	1.64 × 10^−5^	0.0179	MEK inhibitor	[81]
NSCLC	*REV1*	AKT inhibitor VIII^a^	121	0.373	2.51 × 10^−5^	0.0262	AKT inhibitor	[36]
NSCLC	*REV1*	Embelin^a^	121	0.366	3.61 × 10^−5^	0.0349	XIAP inhibitor	[36]
NSCLC	*REV1*	Trametinib^a^	121	0.361	4.71 × 10^−5^	0.0436	MEK inhibitor	[84]
NSCLC	*REV1*	AZD6482^a^	130	0.348	4.84 × 10^−5^	0.0436	PI3Kβ inhibitor	[36]
NSCLC	*REV1*	PD-0332991^a^	100	0.392	5.41 × 10^−5^	0.0471	Palbociclib; CDK 4/6 inhibitor	[100]
PRAD	*REV1*	NSC-207895^a^	5	1.000	1.40 × 10^−24^	1.75 × 10^−21^	MDMX inhibitor	[105]
PRAD	*REV1*	Piperlongumine^a^	5	1.000	1.40 × 10^−24^	1.75 × 10^−21^	Piplartine; ROS induction	[36]
PRAD	*UNG*	ZM-447439^a^	5	1.000	1.40 × 10^−24^	1.75 × 10^−21^	Aurora kinase inhibitor	[103]
PRAD	*UNG*	NU-7441^a^	5	1.000	1.40 × 10^−24^	1.75 × 10^−21^	DNA-PK inhibitor	[36]
PRAD	*UNG*	CCT007093^a^	5	− 1.000	1.40 × 10^−24^	1.75 × 10^−21^	PPM1D inhibitor	[36]
PRAD	*UNG*	JQ1^a^	5	− 1.000	1.40 × 10^−24^	1.75 × 10^−21^	BET inhibitor	[78]
PRAD	*FHIT*	NVP-BHG712^a^	5	− 1.000	1.40 × 10^− 24^	1.75 × 10^−21^	EphB4 inhibitor	[36]
CESC	*FHIT*	MK-2206^a^	5	− 1.000	1.40 × 10^−24^	1.75 × 10^−21^	AKT inhibitor	[36]
MEL	*FHIT*	TAE684^b^	38	0.621	3.24 × 10^−5^	0.0325	ALK inhibitor	[36]
SCLC	*FHIT*	ABT-869^a^	6	− 1.000	< 4.95 × 10^−324^	< 4.95 × 10^−324^	Linifanib; VEGFR/PDGFR family receptor inhibitor	[106]
SCLC	*FHIT*	Mitomycin C^a^	6	− 1.000	< 4.95 × 10^−324^	< 4.95 × 10^−324^	DNA cross-linking/monoalkylating agent	[36, 107]
Pan-cancer	*APOBEC3B*	17-AAG^a^	536	− 0.293	4.25 × 10^−12^	5.85 × 10^−9^	HSP90 inhibitor	[85]

Shown are statistically significant correlations satisfying |*ρ*| > 0.25, *p*_adj_ < 0.05. The *p* values were adjusted for false discovery rate accounting for 5 genes and 255 agents with 275 drug sensitivity measures from CCLE or GDSC resources (*N*_tests_ = 1375 for pan-cancer analysis). Among individual cancer categories, FDR adjustment also accounted for 26 cancer categories with ≥ 5 available cell lines in each category with both gene expression and drug sensitivity data for correlation analysis (*N*_tests_ = 26,110). Abbreviations of cancer categories are provided in the legend of Table 1

*n* sample size for correlation analysis, *ρ* Spearman correlation coefficient, *p p* value prior to FDR adjustment, *p*_*adj*_ FDR-adjusted *p* value, *BET* bromodomain and extraterminal family of proteins, *BRAF* v-raf murine sarcoma viral oncogene homolog B, *CDK* cyclin-dependent kinase, *DNA-PK* DNA-dependent protein kinase, *HDAC* histone deacetylase, *HSP90* molecular chaperone heat shock protein 90, *MEK* mitogen-activated protein kinase kinases, *NAE* NEDD8-activating enzyme E1, *PI3K* phosphatidylinositol-3-kinase, *ROS* reactive oxygen species, *XIAP* X-linked inhibitor of apoptosis

^a^Drug sensitivity data from GDSC [30, 35]

^b^Drug sensitivity data from Cancer Cell Line Encyclopedia (CCLE) [33]

**Fig. 4:**
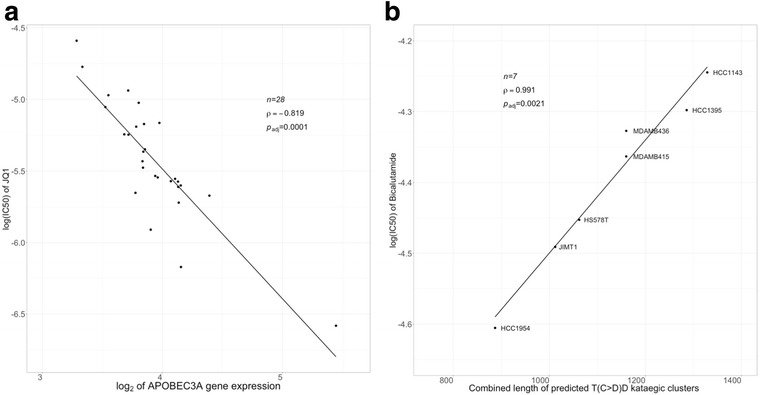
Scatterplots of drug sensitivity measures from the GDSC dataset in selected cancer types. **a** log(IC50) of JQ1 vs log_2_ of the *APOBEC3A* gene expression in pancreatic adenocarcinoma cell lines. **b** log(IC50) of bicalutamide vs the combined length of predicted 5/1000 kataegis clusters with the T(C>D)D motif in breast cancer cell lines. The names of individual breast cancer cell lines are shown. *r* Pearson’s correlation coefficient

In pancreatic adenocarcinoma (PAAD) cell lines, both *APOBEC3A* and *UNG* expression was significantly negatively correlated (Table 6; *ρ* ≤ − 0.819, *p*_adj_ ≤ 0.0001; *n* = 28 for *APOBEC3A* and 5 for *UNG*; *N*_tests_ = 26,610) with log(IC50) of the BET inhibitor JQ1 (Fig. 4a). JQ1 has been reported to inhibit pancreatic cancer cells in vitro and in vivo [76–78]. Correlation of *APOBEC3A* and *UNG* expression with PAAD sensitivity to JQ1 may suggest a possibility that expression of both of these genes may be relevant to the strength of the clinical response to this agent.

Expression of *REV1* in the non-small cell lung cancer cell lines was significantly positively correlated with log(IC50) of MEK (mitogen-activated protein kinase) inhibitors PD-0325901, RDEA119, and trametinib, as well as AKT inhibitor VIII, XIAP inhibitor embelin, PI3Kβ inhibitor AZD6482, and a cyclin-dependent kinase (CDK) 4/6 inhibitor PD-0332991, or palbociclib (Table 6; 0.348 ≤ *ρ* ≤ 0.405, *p*_adj_ ≤ 0.0436, *n* ≥ 100, *N*_tests_ = 26,610). A number of these agents, e.g., trametinib and its combination with palbociclib, have been used or are under investigation for treatment of NSCLC [79, 80]. PD-0325901 has an in vitro inhibiting effect in NSCLC; however, a phase II clinical trial of that antitumor agent in NSCLC patients did not meet the primary efficacy end point [81, 82]. RDEA119 (refametinib) has antitumor activity in a variety of cancer types including in vitro activity in NCSLC, and it has been under evaluation for its effectiveness in NSCLC [82–84].

In melanoma cell lines, *FHIT* expression was associated with chemoresistance to the ALK inhibitor TAE684 (Table 6; *ρ* = 0.621, *p*_adj_ = 0.0326, *n* = 38, *N*_tests_ = 26,610).

Multiple strong significant correlations between expression levels of each of the five candidate genes and sensitivity to multiple agents were found in prostate adenocarcinoma (Table 6); however, the sample size of the PRAD category was small (*n* = 5), and therefore the validity of such correlations may require confirmation in a larger dataset. Similarly, additional correlations found in MM, SAR, CESC, RCC, and SCLC cell lines reported in Table 6 had *n* between 5 and 6 and also require a follow-up confirmation in larger datasets.

In agreement with an earlier report [31], we did not observe an association between *APOBEC3B* expression in breast cancer cell lines and sensitivity to CHK1 inhibitors AZD7762 (*ρ* = − 0.198, *p*_adj_ = 0.8660, *n* = 33, *N*_tests_ = 26,610) or Calbiochem 681,640 (*ρ* = 0.143, *p*_adj_ = 0.933, *n* = 40, *N*_tests_ = 26,610, data not shown), and no other correlations between gene expression and log(IC50) in breast cancer cell lines were statistically significant. Although an association between *APOBEC3B* expression in breast cancer cells and sensitivity to another CHK1 inhibitor, CCT244747, was previously reported [29], that agent was absent from both the CCLE and the GDSC drug sensitivity data sets.

In the pan-cancer analysis, *APOBEC3B* expression was significantly negatively correlated with sensitivity to an HSP90 (molecular chaperone heat shock protein 90) inhibitor 17-AAG (tanespimycin) (Table 6; *ρ* = − 0.293, *p*_adj_ = 5.85 × 10^−9^, *n* = 536, *N*_tests_ = 1375). Higher levels of *APOBEC3B* expression were associated with higher sensitivity to this agent, which may have a clinical significance. 17-AAG acts in a variety of tumor types [85], and sensitivity to this agent was also correlated with *APOBEC3B* in an earlier analysis of RNA-seq gene expression in the CCLE and GDSC cell lines by Cescon and Haibe-Kains [31].

Some other strong association results did not reach statistical significance, but they had *p*_adj_ close to 0.05. For example, higher level of expression of *APOBEC3B* in glioma was correlated with increased sensitivity to an HSP90 inhibitor AUY922 (*ρ* = − 0.556, *p*_adj_ = 0.0701, *n* = 44, *N*_tests_ = 26,610; data not shown). This correlation may have a clinical significance, as this agent has an antitumor effect in glioblastoma [85].

### Correlation between the prevalence of kataegis clusters and chemosensitivity

We examined correlations between chemosensitivity to anticancer drugs and the prevalence of predicted kataegis clusters of APOBEC-like motifs which were identified using the 5/1000 criterion. None of the correlations achieved statistical significance in the combined analysis of all cancer cell lines (*p*_adj_ > 0.1 for comparisons). In a stratified analysis among cancer types, a number of statistically significant strong correlations (0.991 ≤ |*ρ*| ≤ 1.0, *p*_adj_ ≤ 0.0021) were observed in BREAST, COAD/READ, GLIOMA, OVARIAN, and PAAD cell lines (Table 7). However, the number of cell lines in each cancer category with significant correlations was small (*n* = 5–7), and therefore, these correlations need future confirmation in larger collections of cell lines of their respective cancer categories. Among notable correlations, the combined length of clusters with the T(C>D)D motif had a strong correlation (5 ≤ *n* ≤ 7, *N*_tests_ = 1834) with chemoresistance to bicalutamide, a nonsteroidal antiandrogen drug, in the pancreatic adenocarcinoma and breast cancer cell lines (Table 7; Fig. 4b). As discussed above, we did not observe a statistically significant correlation between expression of any candidate gene and the prevalence of T(C>D)D or any other motif in breast cancer cell lines. Sequence variation of breast cancer genomes is shaped by a diversity of mutational processes [86], and further investigation is needed to establish whether the T(C>D)D motif in the breast cancer cell lines is predominantly generated by APOBEC3B and APOBEC3A and/or requires an additional role or REV1, UNG, and FHIT, or whether it involves other molecular mechanisms. Bicalutamide is effective in androgen receptor (AR)-positive breast tumors [87, 88]. Previous studies demonstrated the effectiveness of this agent in triple negative breast tumors [89]. To our knowledge, no relationship between the abundance of APOBEC-like signatures and sensitivity to this agent has been reported, although HER2-enriched cell lines have been reported to have high levels of APOBEC mutagenesis and to be among the breast cancer categories that are likely to be sensitive to bicalutamide [6, 62, 89]. Consistent with an earlier report that suggested the higher prevalence of APOBEC signature in TNBC cells [62], we found that the two TNBC lines with available WES data and bicalutamide sensitivity measures, HCC1395 and MDA-MB-436, had large values of the combined length of the kataegis clusters with the T(C>D)D motif (Fig. 4b). However, both of these cell lines had relatively low sensitivity to bicalutamide in the GDSC dataset (Fig. 4b). We did not find any obvious association between molecular subtypes of the available breast cancer cell lines in our dataset, including their HER2 status [51–53], that could explain the inverse relationship between the length of the T(C>D)D motif clusters and bicalutamide sensitivity presented in Fig. 4b. It is possible that AR-positive status which is associated with bicalutamide sensitivity could affect the expression of genes involved in T(C>D)D motif signature generation; however, the exact molecular mechanisms underlying this relationship remain unclear.

**Table 7:** Significant correlations between the measures of prevalence of APOBEC-like motifs or kataegis clusters and drug sensitivity

Motif	Measure	Agent	*n*	*ρ*	*p*	*p*_adj_	Cancer type
T(C>K)W	Total number of motifs	WZ3105	5	1.000	1.40 × 10^−24^	5.15 × 10^−22^	OVARIAN
T(C>K)W	Total number of motifs	XMD15-27	5	−1.000	1.40 × 10^−24^	5.15 × 10^−22^	OVARIAN
T(C>K)W	Total number of motifs	Tipifarnib	5	1.000	1.40 × 10^−24^	5.15 × 10^−22^	PAAD
T(C>K)W	Total number of motifs	AKT inhibitor VIII	5	−1.000	1.40 × 10^−24^	5.15 × 10^−22^	PAAD
T(C>K)W	Total number of motifs	GSK-1904529A	5	1.000	1.40 × 10^−24^	5.15 × 10^−22^	PAAD
T(C>D)R	Total number of motifs	rTRAIL	6	−1.000	< 4.95 × 10^−324^	< 4.95 × 10^−324^	OVARIAN
T(C>D)R	Total number of motifs	WZ3105	5	1.000	1.40 × 10^−24^	3.22 × 10^−22^	OVARIAN
T(C>D)R	Total number of motifs	XMD15-27	5	−1.000	1.40 × 10^−24^	3.22 × 10^−22^	OVARIAN
T(C>D)R	Total number of motifs	KIN001-266	5	−1.000	1.40 × 10^−24^	3.22 × 10^−22^	COAD/READ
T(C>D)R	Total number of motifs	BMS-536924	5	−1.000	1.40 × 10^−24^	3.22 × 10^−22^	GLIOMA
T(C>D)R	Total number of motifs	HG-5-113-01	5	1.000	1.40 × 10^−24^	3.22 × 10^−22^	BREAST
T(C>D)R	Total number of motifs	Vismodegib	5	−1.000	1.40 × 10^−24^	3.22 × 10^−22^	PAAD
T(C>D)R	Total number of motifs	FH535	5	−1.000	1.40 × 10^−24^	3.22 × 10^−22^	PAAD
T(C>D)D	Total number of motifs	rTRAIL	6	−1.000	< 4.95 × 10^−324^	< 4.95 × 10^−324^	OVARIAN
T(C>D)D	Total number of motifs	WZ3105	5	1.000	1.40 × 10^−24^	3.22 × 10^−22^	OVARIAN
T(C>D)D	Total number of motifs	XMD15-27	5	−1.000	1.40 × 10^−24^	3.22 × 10^−22^	OVARIAN
T(C>D)D	Total number of motifs	NVP-BEZ235	5	−1.000	1.40 × 10^−24^	3.22 × 10^−22^	COAD/READ
T(C>D)D	Total number of motifs	T0901317	5	−1.000	1.40 × 10^−24^	3.22 × 10^−22^	COAD/READ
T(C>D)D	Total number of motifs	RDEA119	5	−1.000	1.40 × 10^−24^	3.22 × 10^−22^	COAD/READ
T(C>D)D	Total number of motifs	HG-5-113-01	5	1.000	1.40 × 10^−24^	3.22 × 10^−22^	BREAST
T(C>D)D	Total number of motifs	LY317615	5	1.000	1.40 × 10^−24^	3.22 × 10^−22^	PAAD
T(C>D)D	Length of kataegis regions	PF-4708671	6	1.000	< 4.95 × 10^−324^	< 4.95 × 10^−324^	BREAST
T(C>D)D	Length of kataegis regions	EX-527	5	−1.000	1.40 × 10^−24^	2.15 × 10^−22^	COAD/READ
T(C>D)D	Length of kataegis regions	KIN001-236	5	−1.000	1.40 × 10^−24^	2.15 × 10^−22^	COAD/READ
T(C>D)D	Length of kataegis regions	CAL-101	5	−1.000	1.40 × 10^−24^	2.15 × 10^−22^	COAD/READ
T(C>D)D	Length of kataegis regions	Y-39983	5	−1.000	1.40 × 10^−24^	2.15 × 10^−22^	COAD/READ
T(C>D)D	Length of kataegis regions	KIN001-270	5	−1.000	1.40 × 10^−24^	2.15 × 10^−22^	COAD/READ
T(C>D)D	Length of kataegis regions	Ruxolitinib	5	−1.000	1.40 × 10^−24^	2.15 × 10^−22^	COAD/READ
T(C>D)D	Length of kataegis regions	XMD14-99	5	−1.000	1.40 × 10^−24^	2.15 × 10^−22^	COAD/READ
T(C>D)D	Length of kataegis regions	QL-VIII-58	5	1.000	1.40 × 10^−24^	2.15 × 10^−22^	BREAST
T(C>D)D	Length of kataegis regions	Genentech Cpd 10	5	1.000	1.40 × 10^−24^	2.15 × 10^−22^	PAAD
T(C>D)D	Length of kataegis regions	Gemcitabine	5	1.000	1.40 × 10^−24^	2.15 × 10^−22^	PAAD
T(C>D)D	Length of kataegis regions	Bicalutamide	5	1.000	1.40 × 10^−24^	2.15 × 10^−22^	PAAD
T(C>D)D	Length of kataegis regions	Bicalutamide	7	0.991	1.46 × 10^−5^	0.0021	BREAST

Shown are statistically significant correlations satisfying *p*_adj_ < 0.05. The *p* values were adjusted for false discovery rate accounting for 4 measures of abundance of each motif category, 255 agents with 275 drug sensitivity measures, and 26 cancer categories with ≥ 5 available cell lines (*N*_tests_ between 1358 and 1874). Drug sensitivity data for all significant correlations listed in the table were obtained from GDSC [30, 35]. Abbreviations of cancer categories are provided in the legend to Table 1

*n* sample size for correlation analysis, *ρ* Spearman correlation coefficient, *p p* value prior to FDR adjustment, *p*_*adj*_ FDR-adjusted *p* value

Multiple other strong correlations were observed in different cancer categories. For example, in pancreatic adenocarcinoma cell lines, log(IC50) values of tipifarnib, a farnesyl transferase inhibitor of the Ras pathway [90], the AKT kinase inhibitor VIII, and the IGF1R/insulin receptor inhibitor GSK-1904529A [36] were associated (|*ρ*| = 1, *p*_adj_ ≤ 5.15 × 10^−22^, *n* = 5, *N*_tests_ = 1834) with the overall counts of the motif T(C>K)W which is commonly attributed to *APOBEC3B* activity. Similarly, log(IC50) of the hedgehog signaling pathway inhibitor vismodegib [91] and of the PPARγ/PPARδ inhibitor FH535 [36] were associated with the overall counts of the T(C>D)R motif. The overall counts of the T(C>D)D motif were associated with log(IC50) of the PKCB inhibitor LY317615 [36], whereas the length of its predicted kataegis regions was associated with log(IC50) of the Aurora kinase A/B inhibitor Genentech Cpd10, a DNA-damaging agent gemcitabine, and, as discussed above, with a nonsteroidal antiandrogen agent bicalutamide (Table 7). While the correlation of these motif counts and kataegis measures with drug sensitivity in PAAD is notable, none of the five candidate genes had significantly associated expression with sensitivity to these agents in PAAD cell lines, although, as discussed above, in the NSCLC cell lines, log(IC50) of AKT inhibitor VIII was correlated with *REV1* expression (Table 6; ρ = 0.373, *p*_adj_ = 2.51 × 10^−5^, *n* = 121, *N*_tests_ = 26,610). Further validation of observations presented in Table 7 is needed in larger datasets of specific cancer types.

## Discussion

We observed a bimodal distribution of *APOBEC3B* expression and unimodal distributions of *APOBEC3A*, *REV1*, *UNG*, and *FHIT* in the pan-cancer dataset (Figs. 2a–e)*.* The bimodal distribution of *APOBEC3B* is likely due to several reasons which include previously reported differences in expression levels of this gene among specific cancer types and individual cell lines within specific cancer categories, along with the germline deletion polymorphism that results in the loss the *APOBEC3B* gene in a subset of the samples [7, 11, 17, 43, 58, 92]. The bimodal distribution of *APOBEC3B* expression is of interest since some studies previously suggested the utility of the genes with bimodally distributed expression patterns as diagnostic and prognostic biomarkers within specific cancer types [93, 94].

We observed low expression levels of *APOBEC3B* in a subset of cell lines and of *APOBEC3A* in many cell lines (Fig. 2; Table 1). Low pre-treatment levels of *APOBEC3A* have been reported previously, and expression of both *APOBEC3B* and *APOBEC3A* has been reported to increase in response to cancer cell treatment with DNA-damaging agents or as part of cellular interferon-induced transcriptional response to viral infections [7]. Low expression levels of *APOBEC3A* in nearly all cancer categories and of *APOBEC3B* in specific cancer categories may provide high levels of noise in correlation analyses [95], and therefore, association results for these genes should be interpreted with caution.

As shown in Fig. 2f, a strong correlation between *APOBEC3A* and *APOBEC3B* expression levels (Table 2) appeared to be independent from the *APOBEC3B* deletion polymorphism which removes the coding area of the *APOBEC3B* gene and creates a fusion transcript of *APOBEC3A* with the 3′-UTR of the *APOBEC3* gene, although earlier reports suggest that this transcript increases *APOBEC3A* levels due to the increase in stability of the fusion transcript [7, 17, 26]. According to Fig. 2f, the correlation between the *APOBEC3A* and *APOBEC3B* gene expression levels also appears to be independent of the copy number status of the *APOBEC3B* gene. One possible explanation could be a transcriptional co-regulation of these two genes, which are located in proximity of one another in the chromosomal region 22q13.1 [7].

Mutagenesis in cancer cells generated due to the activity of APOBEC family members, and in particular of APOBEC3B, has been a subject of many recent studies. While the contributing role of REV1, UNG, and FHIT activity to mutagenic processes has been well established [8, 9, 14, 20, 24, 66], their contribution to the generation of signatures attributed to APOBEC3B and other APOBEC family members and their possible effects on sensitivity to drug treatment have not been examined in depth. Our analysis of cancer cell lines showed that expression levels of *REV1* and *UNG* were significantly correlated with mutagenesis in sarcoma and melanoma cell lines, respectively (Table 5), and that expression of all the five genes examined in our study was significantly correlated with chemosensitivity to various antitumor agents (Table 6).

We focused our analyses on two members of the AID/APOBEC family, APOBEC3A and APOBEC3B, and on three additional genes which are involved in molecular pathways associated in their mutagenesis. Several other APOBEC family members have been implicated in mutagenic processes, with some of them, e.g., AID, APOBEC3F, and APOBEC3G, showing sequence specificities that are distinct from APOBEC3A and APOBEC3B [9, 10, 16, 96]. However, the full extent of overlap among sequence specificities of different APOBEC family members remains an active research area. While we found an increased number of APOBEC-like motifs in mature B cell lymphoma, we did not include the *AID* gene expression in our analysis because both the mutational sequence specificity of AID and the biological context in which AID mutations occur are different from those of APOBEC3B and APOBEC3A [1, 9, 10, 16]. AID is an important deaminating factor in antigen-dependent antibody diversification process of immunoglobulin (Ig) genes through somatic hypermutation and class-switch recombination, and it has also been suggested to be involved in epigenetic processes of demethylation by deaminating cytosine, 5-methylcytosine (5-mC), or 5-hmC [1, 9, 10, 16, 67]. While translocations involving the Ig genes in B cell lymphomas and off-target hypermutational activity of AID in other genome regions have been found in several other cancer types (e.g., gastric, liver, breast, ovarian, lung, and T cell lymphomas), AID-specific mutational patterns are clearly distinguishable from the APOBEC3B/A signature patterns [9, 10]. AID deaminates cytosines within the characteristic WRC motif, or more broadly the WRCY/RGYW motif, with several other AID motif variants having been reported [1, 9, 10, 16]. The AID-specific motif is different from the three motifs reported for APOBEC3B and APOBEC3A that were analyzed in our study, and AID signature patterns can be distinguished computationally from those of APOBEC3A and APOBEC3B [10, 11]. For that reason, we excluded *AID* gene expression from our analysis.

Cancer cell lines provide a convenient model for a combined analysis of molecular information and drug response to a wide range of antitumor agents which cannot be achieved in a clinical setting. However, additional factors may affect clinical outcomes in vivo, including, for example, the strength of the immune response and interaction of the tumor with surrounding tissues. Expression levels of *APOBEC3A*, *APOBEC3B*, *APOBEC3D*, *APOBEC3G*, and *APOBEC3H* in tumor specimens from cancer patients were associated with varying clinical responses to chemotherapy and with overall patient survival, and possible suggested mechanisms of such associations, which may also involve other *APOBEC* genes, include immune targeting of increased mutation diversity due to higher levels of APOBEC mutagenesis, associated inflammation, PD-L1 expression on tumor-infiltrating mononuclear cells, and the degree of T lymphocyte infiltration [7, 92, 97–99].

Because our study analyzed cell line data, it could examine only cell line response to chemotherapy and did not account for in vivo effects that may also influence therapy response. Several correlations of *APOBEC3B* and *APOBEC3A* expression and of motifs attributed to APOBEC3 activity observed in our study were consistent with drug sensitivity associations with *APOBEC3A* and *APOBEC3B* activity identified in cell line models by a previous study [31]. Our analysis of breast cancer cell lines, however, was not able to replicate the previously reported correlation of *APOBEC3B* expression level in vivo with resistance to tamoxifen in a clinical setting or in murine xenograft models in ER^+^ breast cancer [18] due to the lack of statistical significance. We observed *ρ* between − 0.118 and − 0.049, *p*_adj_ > 0.94 (*n* = 43, *N*_tests_ = 26,110) for correlations of both *APOBEC3B* and *APOBEC3A* expression levels with log(IC50) of tamoxifen in breast cancer cell lines. Stratified analysis of ER^−^ and ER^+^ breast cell lines with available information about their estrogen receptor status showed the absence of association in the ER^−^ cell lines with log(IC50) of tamoxifen (− 0.083 ≤ *ρ* ≤ − 0.026, unadjusted *p* > 0.67, *n* = 28). In the ER^+^ cell lines, we observed an association with sensitivity to tamoxifen for both genes (*ρ* = 0.− 0.362 for *APOBEC3A* and − 0.418 for *APOBEC3B*, *n* = 13) which was consistent with that of Law et al. [18]; however, the results for both genes in our study were statistically non-significant (*p* = 0.157 for *APOBEC3A* and 0.224 for *APOBEC3B*), possibly due to a small number of ER^+^ breast cell lines in the dataset. Additionally, the study of Law et al. [18], which reported association of the *APOBEC3B* expression with tamoxifen resistance, included primary breast tumors from hormone therapy-naïve patients, whereas some of the cell lines in our analysis were likely obtained from patients with prior treatment. In our study, none of the correlations of chemosensitivity to tamoxifen with expression of either of the five candidate genes in any cancer category or in the pan-cancer analysis achieved statistical significance. Therefore, while our use of cell line resources was able to draw from a wealth of molecular information and the data on sensitivity to multiple tumor agents, in using the cell line-based approach, we also encountered several limitations including restricted clinical information, much smaller sample sizes than those available for patient-based clinical studies, and the absence of normal tissues from the same patients that could allow for more accurate inference of mutation calls and for tissue-specific normalization of gene expression levels.

Despite these limitations, we observed a number of correlations, e.g., those between *APOBEC3A* and *APOBEC3B* expression levels, that have also been reported in patient tumor samples [7]. In addition, our results presented in Table 6 show that expression of all five candidate genes was correlated with sensitivity to chemotherapy and that log(IC50) of a number of antitumor agents was significantly correlated not only with expression levels of *APOBEC3B*, but also with those of *APOBEC3A*, *REV1 UNG*, and *FHIT*. Three of these genes, *REV1*, *UNG*, and *APOBEC3A*, were also associated with overall mutation activity and/or with prevalence of APOBEC-like motifs and kataegis clusters in specific cancer types. Because APOBEC3A is also involved in RNA editing [26], association of its expression with drug sensitivity might potentially involve the RNA editing mechanism instead of or in addition to DNA mutagenesis; however, both of these mechanisms would require additional experimental validation. Additionally, as APOBEC3A has also been linked to epigenetic processes of DNA demethylation [1, 3, 4], its involvement in epigenetic mechanisms of sensitivity or resistance to cancer treatment cannot be ruled out, even though the associations reported in Tables 6 and 7 involve non-epigenetic agents.

Recent studies suggest that clustered mutations, including those attributed to APOBEC activity, more accurately represent mutagenic processes in tumors than do overall mutation rates [13]. We observed significant correlations of the prevalence of all the three APOBEC-like motifs with chemosensitivity to multiple agents in small groups of cell lines from specific cancer types (Table 7). When using measures of kataegis clusters, we observed correlations of the combined length of kataegis clusters of the least specific T(C>D)D motif with sensitivity to various agents in breast, pancreatic adenocarcinoma, and colon adenocarcinoma and rectum adenocarcinoma cancer cell lines. However, because expression of none of the five candidate genes was significantly associated with the abundance of the T(C>D)D motif or with the clusters containing this motif, further studies are needed to better understand the mutational pathways generating the T(C>D)D motif and to examine whether additional members of the APOBEC family or translesion DNA polymerases may contribute to its occurrence. Molecular mechanisms underlying correlations of cell line response to treatment with specific agents with motif abundance or with expression of *APOBEC3A*, *APOBEC3B*, *REV1*, *UNG*, and *FHIT* also require further investigation. Nevertheless, specific correlations observed in our studies suggest that both expression levels of candidate genes and the prevalence of APOBEC-like motifs and their clusters could potentially be examined for their roles as biomarkers of drug sensitivity to several agents. Association of activity of these genes with drug response could be examined further when significantly associated agents are evaluated in experimental in vitro studies and in a clinical setting.

## Conclusions

Our analysis of cancer cell line data identified associations of drug sensitivity with expression levels of *APOBEC3A*, *APOBEC3B*, *REV1*, and *UNG* genes and with abundance of sequence motifs and kataegis clusters attributed to APOBEC activity. The analysis of exome sequence data suggested that expression of *REV1* and *UNG* and to a lesser extent of *APOBEC3A* was correlated with mutation patterns attributed to APOBEC activity, suggesting that APOBEC-like mutagenic patterns may result from the complex interplay among multiple molecular factors. Future studies may examine the biological mechanisms that could explain how each of the five genes associated with APOBEC-like mutagenic processes may contribute to sensitivity or resistance of tumor cells to cancer drug treatment.

## Abbreviations

5-hmC: 5-Hydroxymethyl-cytozine
5-hmU: 5-Hydroxymethyl-uracil
5-mC: 5-Methylcytosine
ALL: Acute lymphocytic leukemia
APOBEC: Apolipoprotein B mRNA-editing enzyme, catalytic polypeptide-like
BET: Bromodomain and extraterminal family of proteins
BLADDER: Bladder cancer (including the TCGA category of bladder urothelial carcinoma and other types of bladder cancer)
BRAF: V-raf murine sarcoma viral oncogene homolog B
BREAST: Breast cancer (including the TCGA category of breast invasive carcinoma and other types of breast carcinomas)
CCLE: Cancer Cell Line Encyclopedia
CDK: Cyclin-dependent kinase
CESC: Cervical squamous cell carcinoma and endocervical adenocarcinoma
CLLE: Chronic lymphocytic leukemia
COAD/READ: Colon adenocarcinoma and rectum adenocarcinoma
DA: Duodenal adenocarcinoma
DNA-PK: DNA-dependent protein kinase
EC: Esophageal cancer (including esophageal carcinoma and Barrett adenocarcinoma)
ER^−^: Estrogen receptor-negative
ER^+^: Estrogen receptor-positive
FDR: False discovery rate
FHIT: Fragile histidine triad protein
GDSC: Genomics of Drug Sensitivity in Cancer
GLIOMA: Glioma brain tumors (including astrocytoma, lower-grade glioma, and glioblastoma multiforme)
HDAC: Histone deacetylase
HNSC: Head and neck squamous cell carcinoma
HSP90: Molecular chaperone heat shock protein 90
Ig: Immunoglobulin
IGF1R: Insulin-like growth factor 1 receptor
IR: Insulin receptor
LAML: Acute myeloid leukemia
LCML: Chronic myelogenous leukemia
LIHC: Liver hepatocellular carcinoma
MATBCL: Mature B cell lymphoma (including lymphoid neoplasm diffuse large B cell lymphoma, Burkitt lymphoma, and other categories)
MB: Medulloblastoma
MEK: Mitogen-activated protein kinase kinase
MEL: Melanoma
MEN: Meningioma
MESO: Mesothelioma
MGCT: Malignant giant cell tumor of bone
MISC: Miscellaneous categories of cancer including rare cancers or cancers with unspecified information
MM: Multiple myeloma
NAE: NEDD8-activating enzyme E1
NCI: National Cancer Institute
NSCLC: Non-small cell lung cancer (including also lung adenocarcinoma and lung squamous cell carcinoma)
OVARIAN: Ovarian cancer (including the TCGA category of ovarian serous cyctadenocarcinoma and other categories)
PAAD: Pancreatic adenocarcinoma
Pan-cancer: Combined analysis of all cancer categories
PI3K: Phosphatidylinositol-3-kinase
PKCB: Protein kinase C β type
PNET: Primitive neuroectodermal tumors (including neuroblastoma and other categories)
PPAR: Peroxisome proliferator-activated receptor
PRAD: Prostate adenocarcinoma
RCC: Renal cell carcinoma (including kidney clear cell carcinoma, kidney papillary carcinoma, and other categories)
ROS: Reactive oxygen species
SAR: Sarcoma
SCLC: Small cell lung cancer
SD: Standard deviation
STAD: Stomach adenocarcinoma
TCGA: The Cancer Genome Atlas
THCA: Thyroid carcinoma
TNCB: Triple negative breast cancer
UCEC: Uterine corpus endometrial carcinoma
UNG: Uracil-specific uracil DNA glycosylase
WES: Whole-exome sequencing
XIAP: X-linked inhibitor of apoptosis
